# PINCH-1 regulates mitochondrial dynamics to promote proline synthesis and tumor growth

**DOI:** 10.1038/s41467-020-18753-6

**Published:** 2020-10-01

**Authors:** Ling Guo, Chunhong Cui, Jiaxin Wang, Jifan Yuan, Qingyang Yang, Ping Zhang, Wen Su, Ruolu Bao, Jingchao Ran, Chuanyue Wu

**Affiliations:** 1 Guangdong Provincial Key Laboratory of Cell Microenvironment and Disease Research, Shenzhen Key Laboratory of Cell Microenvironment, Academy for Advanced Interdisciplinary Studies and Department of Biology, Southern University of Science and Technology, Shenzhen, China; 2 Department of Pathology, Shenzhen University Health Science Center, Shenzhen, China; 3 Department of Pathology, University of Pittsburgh School of Medicine, Pittsburgh, PA 15261 USA

**Keywords:** Cancer metabolism, Cancer microenvironment, Lung cancer, Focal adhesion

## Abstract

Reprograming of proline metabolism is critical for tumor growth. Here we show that PINCH-1 is highly expressed in lung adenocarcinoma and promotes proline synthesis through regulation of mitochondrial dynamics. Knockout (KO) of PINCH-1 increases dynamin-related protein 1 (DRP1) expression and mitochondrial fragmentation, which suppresses kindlin-2 mitochondrial translocation and interaction with pyrroline-5-carboxylate reductase 1 (PYCR1), resulting in inhibition of proline synthesis and cell proliferation. Depletion of DRP1 reverses PINCH-1 deficiency-induced defects on mitochondrial dynamics, proline synthesis and cell proliferation. Furthermore, overexpression of PYCR1 in PINCH-1 KO cells restores proline synthesis and cell proliferation, and suppresses DRP1 expression and mitochondrial fragmentation. Finally, ablation of PINCH-1 from lung adenocarcinoma in mouse increases DRP1 expression and inhibits PYCR1 expression, proline synthesis, fibrosis and tumor growth. Our results identify a signaling axis consisting of PINCH-1, DRP1 and PYCR1 that regulates mitochondrial dynamics and proline synthesis, and suggest an attractive strategy for alleviation of tumor growth.

## Abstract (web summary)

Proline metabolism is crucial to tumor proliferation. Here, the authors show PINCH-1 deficiency inhibited proline synthesis by promoting mitochondrial fragmentation via DRP1, downregulating PYCR1 expression and reducing cell proliferation in vitro and in vivo.

## Introduction

Proline metabolism is crucial for energy production, redox balance, signaling, and protein synthesis, in particular that of collagen, into which nearly 25% of amino acids incorporated are proline. In diseases with fast proliferating cells such as cancer, the demand for proline synthesis appears to be markedly increased^1^. Indeed, recent studies have shown that the level of proline is markedly altered in cancer^2–5^. Furthermore, PYCR1, a mitochondrial protein that is critical for proline synthesis, is one of the most overexpressed metabolic enzymes in cancer^2–4,6–9^. However, despite the increase of PYCR1 expression, the level of proline is often inadequate for maintaining high level of protein synthesis and redox balance in proliferating cancer cells^2,10^. This “proline vulnerability” may provide an opportunity for inhibition of fibrosis and tumor growth. Thus, it is important to elucidate how PYCR1 and proline synthesis are regulated in cells. We recently found that a fraction of kindlin-2, a focal adhesion protein, is translocated into mitochondria where it forms a complex with PYCR1, which prevents PYCR1 degradation and thereby promotes proline synthesis, tumor fibrosis, and growth^11^. The cellular mechanism by which kindlin-2 translocation into mitochondria and interaction with PYCR1 are regulated, however, remained to be determined.

Mitochondria are dynamic subcellular organelles that are undergoing constant fission and fusion, the balance of which controls both the ultrastructure or morphology and functions of mitochondria^12–17^. Mitochondrial fission provides adequate number of mitochondria during cell division and facilitates proper distribution of mitochondria and removal of damaged segments of mitochondria^12–14^. However, excessive mitochondrial fission may impair mitochondrial functions. Mitochondrial fusion, on the other hand, allows mixing of contents from different mitochondria and maximizing the capacity for mitochondrial functions such as oxidative phosphorylation^13,14,17^. At the molecular level, mitochondrial fission is mediated by DRP1 and its receptors including Mitochondrial Fission Factor (MFF), Fission (FIS1), Mitochondrial Dynamics Protein Of 49 KDa (MID49), and Mitochondrial Dynamics Protein Of 51 KDa (MID51)^12–17^. Mitochondrial fusion, on the other hand, is mediated by Mitofusin 1 (MFN1), Mitofusin 2 (MFN2), and optic atrophy 1 (OPA1)^12–17^. Alterations of mitochondrial dynamics are intimately involved in the pathogenesis and progression of many common diseases including cancer^18–23^. The majority of the proteins found in the mitochondria are encoded by nuclear genes, synthesized in the cytosol, and transported into the mitochondria. Mitochondrial recruitment of proteins from the cytosol is often regulated by mitochondria membrane potential, which is influenced to a great extent by mitochondrial dynamics^24–27^. Alterations of mitochondrial dynamics, therefore, can exert strong effects on mitochondrial recruitment of proteins and metabolic activities^12–17^. Elucidation of the signaling pathways that regulate mitochondrial dynamics is an important research area of current cell biology and medicine.

Cell-extracellular matrix (ECM) adhesion has been implicated in regulation of mitochondrial dynamics and metabolic activities but the underlying molecular mechanisms are not clear^11,28–30^. In this study, we show that PINCH-1, a widely expressed and evolutionally conserved focal adhesion protein^31–45^, acts as an important regulator of mitochondrial fragmentation, proline synthesis, tumor fibrosis, and growth. Ablation of PINCH-1 significantly increases DRP1 expression, resulting in increased mitochondrial fragmentation, decreased kindlin-2 translocation into mitochondria and interaction with PYCR1, diminishes the levels of PYCR1 and proline synthesis, and reduces cell proliferation. Depletion of DRP1 effectively reverses PINCH-1 deficiency-induced effects on mitochondrial fragmentation, kindlin-2 translocation, and interaction with PYCR1, proline synthesis, and cell proliferation. Furthermore, overexpression of PYCR1 in PINCH-1 deficient cells suppresses DRP1 expression and mitochondrial fragmentation and reverses the PINCH-1 deficiency-induced inhibition on proline synthesis and cell proliferation. Finally, we provide in vivo evidence showing that ablation of PINCH-1 from lung adenocarcinoma in mouse increases DRP1 expression and reduces the levels of PYCR1, proline synthesis, collagen matrix, and tumor growth. These findings identify a- PINCH-1-DRP1-PYCR1 signaling axis that is critically involved in regulation of mitochondrial dynamics and proline synthesis, and suggest an attractive strategy for control of tumor fibrosis and growth.

## Results

### PINCH-1 regulates mitochondrial fragmentation

**Fig. 1:**
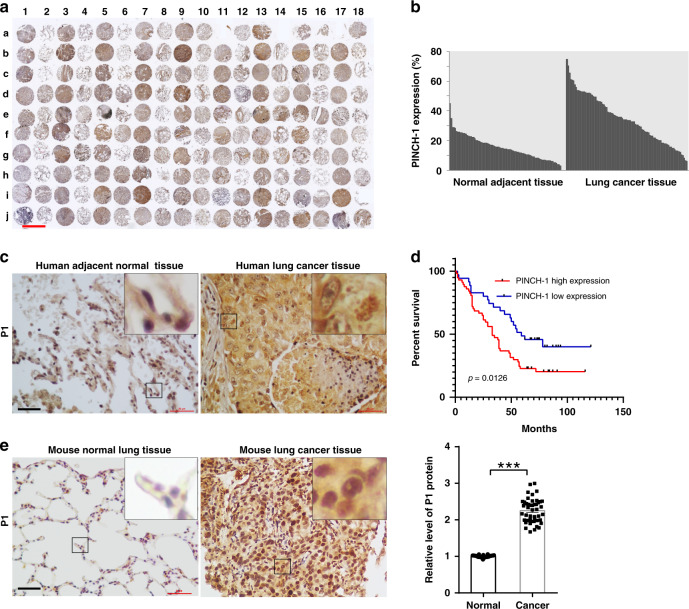
PINCH-1 level is increased in lung adenocarcinoma. **a**, **b** Human lung adenocarcinoma tissues and adjacent normal tissues from a microarray were stained with anti-PINCH-1 (P1) antibody (**a**). The samples in columns 1, 3, 5, 7, 9, 11, 13, 15, and 17 on rows a–i and columns 1, 3, 5, 7, 9, 11, and 13–18 on row j were derived from lung adenocarcinoma tissues. All other samples were derived from adjacent normal tissues. Bar = 1 mm. The mean intensities (arbitrary units) of PINCH-1 staining from lung adenocarcinoma tissues (*n* = 93) and normal adjacent tissues (*n* = 87) were quantified (**b**). **c** Higher magnification images of representative IHC staining shown in panel a obtained with anti-PINCH-1 antibody (brown) and haematoxylin counterstain (blue). Bar = 20μm. The boxed areas in the IHC staining were enlarged and shown in the upper right corner. **d** Kaplan–Meier survival curve of 90 lung adenocarcinoma patients. Patients were divided into two groups according to the mean intensities of PINCH-1 staining in cancer tissues of the tissue array (high expression: *n* = 57, low expression: *n* = 35, Log-rank (Mantel–Cox) test was used for the statistical analysis). **e** Lung adenocarcinoma was induced by administration of Ad-Cre into the lung of Kras^LSL−G12D/+^ mice. Sixteen weeks later, sections from the Kras^LSL−G12D/+^ mice administrated with Ad-Cre or without Ad-Cre as a control were analyzed by immunostaining with anti-PINCH-1 antibody. Bar = 20 μm. The boxed areas in the IHC staining were enlarged and shown in the upper right corner. The mean intensities of PINCH-1 staining in lung adenocarcinoma were quantified and compared to those of normal lung tissue (normalized to 1; normal group *n* = 36 fields from 6 mice, cancer group *n* = 49 fields from 6 mice; *P* < 0.0001) (e, right panel). Data in e represent mean ± SEM. Statistical significance was calculated using two-tailed unpaired Student’s *t*-test, ***P < 0.001. Source data are provided as a Source Data file.

**Fig. 2:**
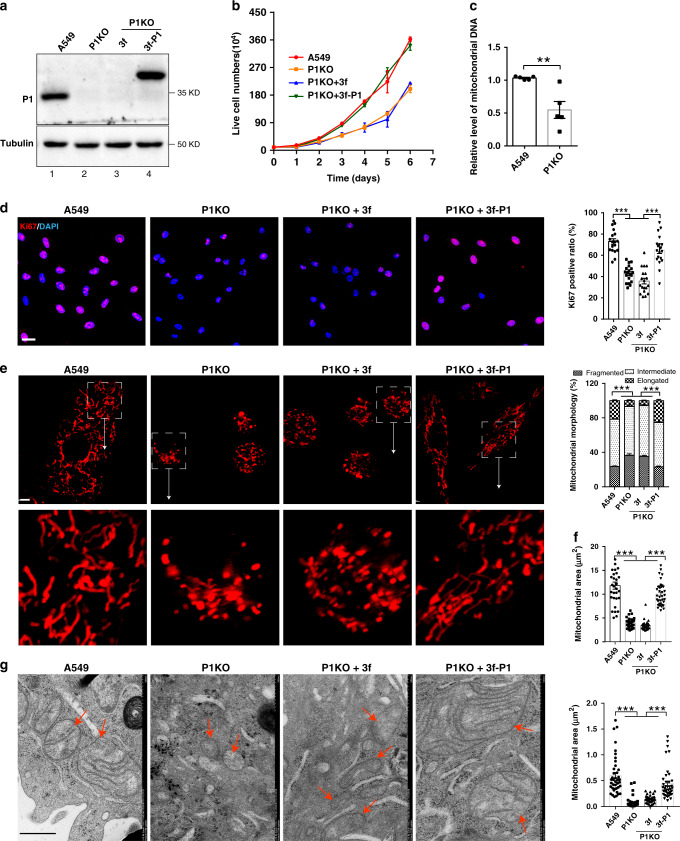
PINCH-1 regulates mitochondrial fragmentation and A549 cancer cell proliferation. PINCH-1 KO (P1KO) A549 cells were infected with lentiviral vectors encoding 3xFLAG-tagged PINCH-1 (3f-P1), or only 3xFLAG vector (3 f) for 3 days. **a** Cells (as indicated) were analyzed by Western blotting with antibodies recognizing PINCH-1 (P1) and tubulin. The samples were derived from the same experiment and the blots were processed in parallel. **b** The numbers of live cells in each well at different time points (as indicated) were analyzed using trypan blue exclusion assay as described in the “Methods” (**b**, *n* = 3). **c** The relative level of mtDNA in the PINCH-1 KO A549 cells was analyzed by RT-PCR as described in the “Methods” and compared to that of wild type A549 cells (normalized to 1; *n* = 5; *P* = 0.0054). **d** The cells were stained with DAPI (blue) and antibody for Ki67 (purple). Bar, 25 μm. The percentages of Ki67-positive cells were quantified (right, A549 or P1KO + 3f *n* = 20 fields, P1KO or P1KO + 3f-P1 *n* = 19 fields; A549 vs P1KO *P* < 0.0001, A549 vs P1KO + 3f *P* < 0.0001, P1KO vs P1 + 3f-P1 *P* < 0.0001, P1KO + 3f vs P1KO + 3f-P1 *P* < 0.0001). **e**, **f** Mitochondria were stained with MitoTracker Red CMXRos (left panels) and the percentages of mitochondria with different morphologies were quantified (right panel, A549 *n* = 80 cells, P1KO *n* = 30 cells, P1KO + 3f *n* = 40 cells, P1KO + 3f-P1 *n* = 39 cells; A549 vs P1KO *P* < 0.0001, A549 vs P1KO + 3f *P* < 0.0001, P1KO vs P1 + 3f-P1 *P* < 0.0001, P1KO + 3f vs P1KO + 3f-P1 *P* < 0.0001). Bar, 5 μm. Mitochondrial areas in z-stack images were quantified (**f**, A549 *n* = 31 cells, P1KO *n* = 35 cells, P1KO + 3f *n* = 36 cells, P1KO + 3f-P1 *n* = 37 cells; A549 vs P1KO *P* < 0.0001, A549 vs P1KO + 3f *P* < 0.0001, P1KO vs P1 + 3f-P1 *P* < 0.0001, P1KO + 3f vs P1KO + 3f-P1 *P* < 0.0001). **g** Mitochondria (red arrows) were observed under TEM and the areas of mitochondria were quantified (right, A549, P1KO or P1KO + 3f-P1 *n* = 40 mitochondria, P1KO + 3f *n* = 52 mitochondria; A549 vs P1KO *P* < 0.0001, A549 vs P1KO + 3f *P* < 0.0001, P1KO vs P1 + 3f-P1 *P* < 0.0001, P1KO + 3f vs P1KO + 3f-P1 *P* < 0.0001). Bar, 500 nm. Data in **b**–**g** represent mean ± SEM. Statistical significance was calculated using one-way ANOVA with Tukey–Kramer post-hoc analysis,**P < 0.01; ***P < 0.001. Source data are provided as a Source Data file.

**Fig. 3:**
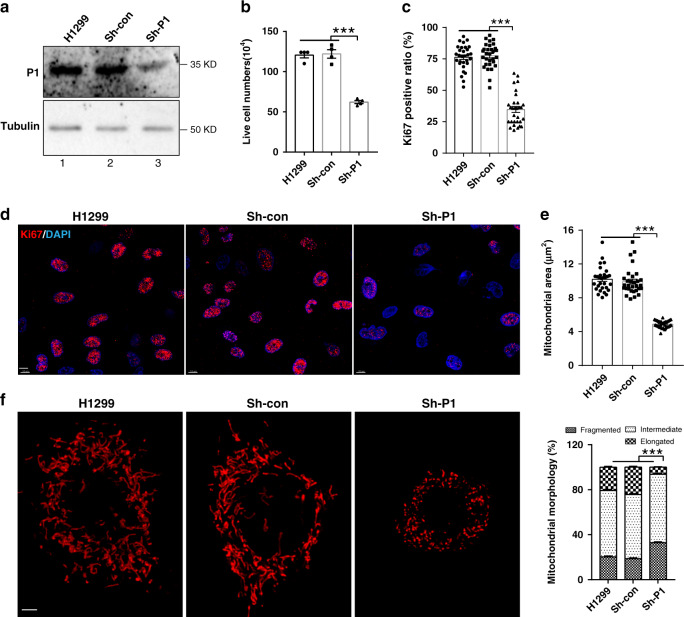
PINCH-1 regulates mitochondrial fragmentation and H1299 cancer cell proliferation. **a** H1299 lung adenocarcinoma cells were infected with PINCH-1 shRNA (Sh-P1) or control RNA (Sh-con) lentivirus, cultured for 5 days and analyzed by Western blotting with antibodies recognizing PINCH-1 (P1) or tubulin. **b** The cells were cultured for 4 days and the numbers of live cells were analyzed as in Fig. 2b (*n* = 4; H1299 vs Sh-P1 *P* < 0.0001, Sh-con vs Sh-P1 *P* < 0.0001). **c**, **d** The cells were stained with DAPI (blue) and antibody for Ki67 (purple) (**d**). Bar, 25μm. The percentages of Ki67-positive cells were quantified (**e**, *n* = 30; H1299 vs Sh-P1 *P* < 0.0001, Sh-con vs Sh-P1 *P* < 0.0001). **e**, **f** Mitochondria were stained with MitoTracker Red CMXRos (**f**, left panels) and the percentages of mitochondria with different morphologies were quantified (**f**, right panel, H1299 *n* = 33 cells, Sh-con *n* = 48 cells, Sh-P1 *n* = 56 cells; H1299 vs Sh-P1 *P* < 0.0001, Sh-con vs Sh-P1 *P* < 0.0001). Bar, 5 μm. Mitochondrial areas in z-stack images were quantified (**e**, *n* = 30 cells; H1299 vs Sh-P1 *P* < 0.0001, Sh-con vs Sh-P1 *P* < 0.0001). Data in **b**, **c**, **e**, and **f** represent mean ± SEM. Statistical significance was calculated using one-way ANOVA with Tukey–Kramer post-hoc analysis. ***P < 0.001. Source data are provided as a Source Data file.

Immunohistochemical (IHC) staining of tissues from human patients with lung adenocarcinoma revealed that PINCH-1 level is significantly increased in lung adenocarcinoma compared to that in normal lung tissues (Fig. 1a–c). Furthermore, higher levels of PINCH-1 are positively correlated with poorer human patient survival (Fig. 1d). A higher level of PINCH-1 was also observed in Kras^G12D^-induced lung adenocarcinoma in mice compared with that in normal lung tissues (Fig. 1e). To investigate the functional significance of elevated PINCH-1 level in lung adenocarcinoma, we knocked out PINCH-1 from A549 lung adenocarcinoma cells (Fig. 2a, compare lane 2 with lane 1) using CRISPR/Cas9-mediated gene editing. Consistent with our previous studies^40^, KO of PINCH-1 reduced the levels of ILK and α-parvin (Supplementary Fig. 1a, compare lane 2 with lane 1). The level of kindlin-2 was not significantly changed in response to loss of PINCH-1 (Supplementary Fig. 1a, compare lane 2 with lane 1). Conversely, KO of kindlin-2 did not significantly alter the levels of PINCH-1, ILK, and α-parvin (Supplementary Fig. 1a, compare lane 4 with lane 3). Consistent with previous studies^40,42,37,39,46^, loss of PINCH-1 reduced cell-ECM adhesion (Supplementary Fig. 1b) and spreading (Supplementary Fig. 1c). Furthermore, KO of PINCH-1 significantly reduced cell proliferation (Fig. 2b, d). Immunofluorescent staining and electron microscopic analyses showed that mitochondria in PINCH-1 KO cells were more fragmented compared with those in control cells (Fig. 2e–g). The level of mitochondrial DNA was also reduced in response to loss of PINCH-1 (Fig. 2c). Re-expression of 3xFLAG-tagged PINCH-1 (3f-P1) in PINCH-1 KO cells (Fig. 2a, lane 4) restored normal elongated tubular structure of mitochondria (Fig. 2e–g) and cell proliferation (Fig. 2b, d). Similarly, knockdown of PINCH-1 from H1299 lung adenocarcinoma cells (Fig. 3a) also increased mitochondrial fragmentation (Fig. 3e, f) and reduced cell proliferation (Fig. 3b–d). Collectively, these results suggest that PINCH-1 is critically involved in regulation of mitochondrial fragmentation and cell proliferation.

### PINCH-1 regulates mitochondrial fragmentation through DRP1

**Fig. 4:**
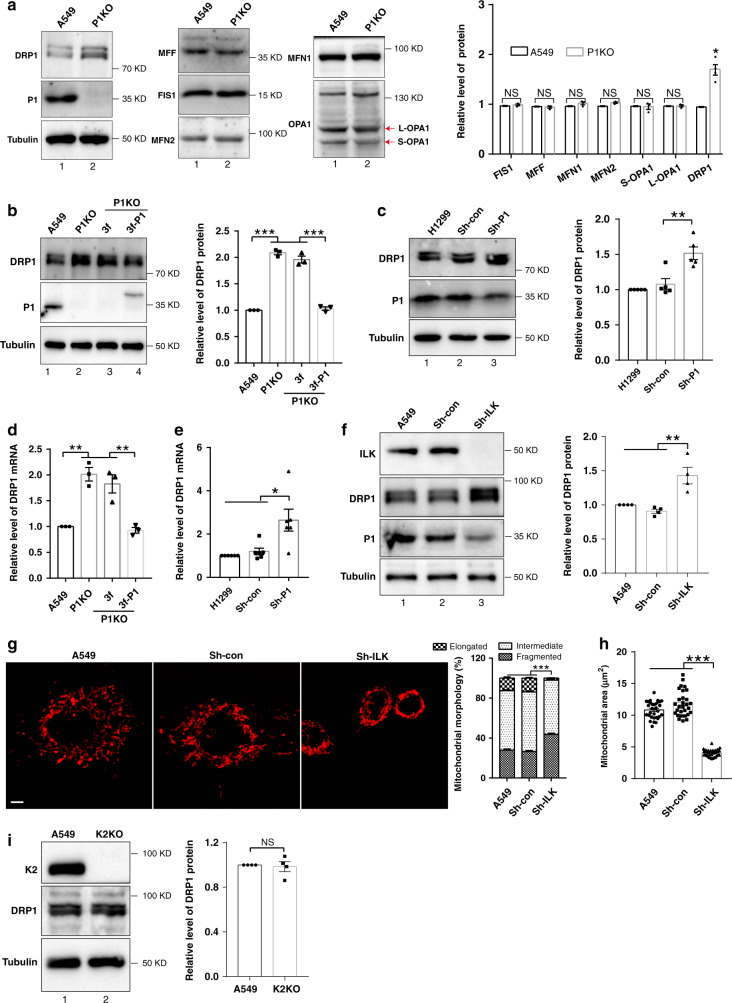
Loss of PINCH-1 promotes DRP1 expression. **a** PINCH-1 KO A549 cells were analyzed by Western blotting with antibodies as indicated. The levels of DRP1, FIS1, MFF, short form of OPA1(S-OPA1), long form of OPA1(L-OPA1), MFN1 and MFN2 in PINCH-1 KO A549 cells were quantified by densitometry and compared to those in A549 cells (normalized to 1) (right, *n* = 3). **b** PINCH-1 KO A549 cells were infected with lentiviral vectors encoding 3f-P1 or 3 f. Three days later, cells (as indicated) were analyzed by Western blotting. DRP1 level in indicated samples was quantified by densitometry and compared to A549 (right, *n* = 3). **c** H1299 cells were infected with Sh-P1 or Sh-con lentivirus and analyzed by Western blotting with antibodies as indicated. The levels of DRP1 in PINCH-1 knockdown H1299 cells were quantified by densitometry and compared to those in H1299 cells (normalized to 1) (right, *n* = 5). **d** DRP1 mRNA levels in A549 cells (as indicated) were analyzed by RT-PCR (*n* = 3). **e** DRP1 mRNA levels in H1299 cells (as indicated) were analyzed by RT-PCR (*n* = 6). **f** A549 cells were infected with ILK shRNA (Sh-ILK) or Sh-con lentivirus and analyzed by Western blotting as indicated. The levels of DRP1 in ILK knockdown cells were quantified by densitometry and compared to those in A549 cells (normalized to 1) (right, *n* = 4). **g** Mitochondria were stained with MitoTracker Red CMXRos and the percentages of mitochondria with different morphologies were quantified (right, *n* = 30 cells). Bar, 5 μm. **h** Mitochondrial areas in z-stack images were quantified (*n* = 31 cells). **i** Wild type and kindlin2 (K2) KO A549 cells were analyzed by Western blotting with antibodies recognizing DRP1, P1 or tubulin. The level of DRP1 in kindlin2 KO A549 cells was quantified by densitometry and compared to that in wild type A549 cells (normalized to 1) (right, *n* = 4). Data represent mean ± SEM. Statistical significance was calculated using one-way ANOVA with Tukey–Kramer post-hoc analysis. *P < 0.05; **P < 0.01; ***P < 0.001; NS no significance. Source data are provided as a Source Data file. The samples in **a**, **b**, **c**, **f**, and **i** were from the same experiment and the blots were processed in parallel.

We next sought to investigate the mechanism by which PINCH-1 regulates mitochondrial fragmentation. Immunofluorescent staining experiments failed to detect PINCH-1 or its associated proteins ILK and α-parvin in the mitochondria (Supplementary Fig. 2), suggesting that PINCH-1 might regulate mitochondrial fragmentation indirectly. To test this, we analyzed the effects of PINCH-1 deficiency on proteins that are critically involved in mitochondrial dynamics. The results showed that both the protein (Fig. 4a, b, compare lane 2 with lane 1) and mRNA (Fig. 4d) levels of DRP1, a key mediator of mitochondrial fission, were markedly increased in PINCH-1 KO A549 cells compared with those in wild type A549 cells. By contrast, loss of PINCH-1 did not significantly change the levels of MFF, FIS1, MFN1, MFN2, and the long and short forms of OPA1 (Fig. 4a). Similar results were obtained when PINCH-1 was knocked down from A549 lung carcinoma cells by RNAi (Supplementary Fig. 3). Knockdown of PINCH-1 from H1299 lung adenocarcinoma cells also increased the protein (Fig. 4c) and mRNA (Fig. 4e) levels of DRP1. Re-expression of 3xFLAG-tagged PINCH-1 in PINCH-1 KO cells restored both the protein (Fig. 4b, compare lane 4 with lane 3) and mRNA (Fig. 4d) levels of DRP1. KO of ILK, which reduced the level of PINCH-1^40^ (Fig. 4f), also increased the DRP1 level (Fig. 4f), resulting in increased mitochondrial fragmentation (Fig. 4g and h). By contrast, KO of kindlin-2, which did not reduce the level of PINCH-1 (Supplementary Fig. 1a), failed to alter the level of DRP1 (Fig. 4i). Collectively, these results suggest that PINCH-1 and ILK but not kindlin-2 are critical for regulation of DRP1 expression.

**Fig. 5:**
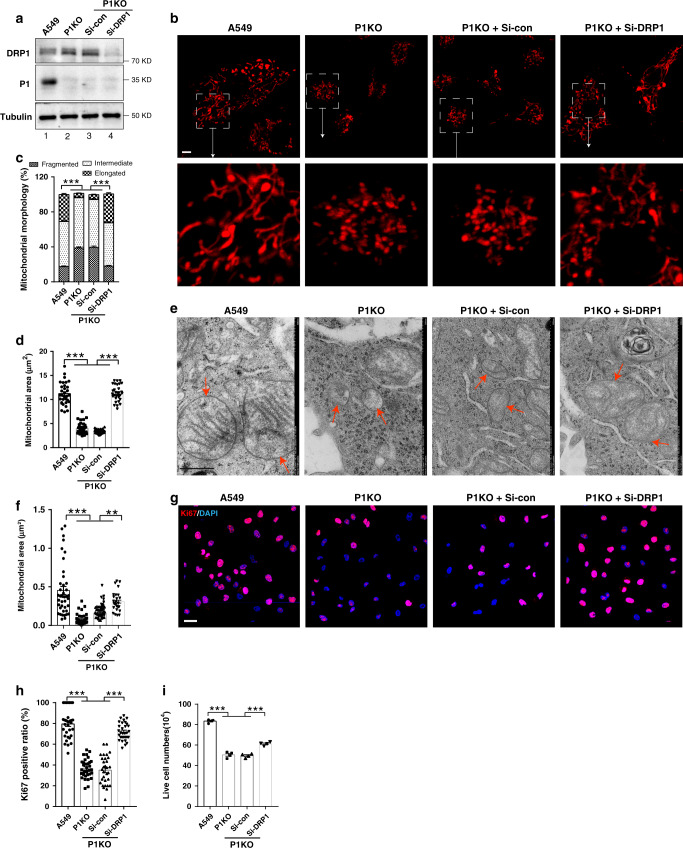
Depletion of DRP1 reverses PINCH-1 KO-induced increase of mitochondrial fragmentation. **a** PINCH-1 KO A549 cells were transfected with siRNA targeting DRP1 (Si-DRP1) or control siRNA (Si-con) as indicated. Three days later, the cells were analyzed by Western blotting with antibodies recognizing DRP1, P1, or tubulin. The samples were from the same experiment and the blots were processed in parallel. Mitochondria were stained with MitoTracker Red CMXRos (**b**) and the percentages of mitochondria with different morphologies were quantified (**c**) (A549 *n* = 43 cells, P1KO *n* = 70 cells, P1KO + Si-Con *n* = 77 cells, P1KO + Si-DRP1 *n* = 55cells; A549 vs P1KO *P* < 0.0001, A549 vs P1KO + Si-con *P* < 0.0001, P1KO vs P1KO + Si-DRP1 *P* < 0.0001, P1KO + Si-con vs P1KO + Si-con P < 0.0001). Bar, 5μm. **d** Mitochondrial areas in z-stack images were quantified (A549 and P1KO and P1KO + Si-con n = 32 cells, P1KO + Si-DRP1 *n* = 30 cells; A549 vs P1KO *P* < 0.0001, A549 vs P1KO + Si-con *P* < 0.0001, P1KO vs P1KO + Si-DRP1 *P* < 0.0001, P1KO + Si-con vs P1KO + Si-con *P* < 0.0001). **e**, **f** Mitochondria (red arrows) were observed under TEM (**e**, bar, 500 nm) and the areas of mitochondria were quantified (**f**, *n* = 40 mitochondria; A549 vs P1KO P < 0.0001, A549 vs P1KO + Si-con P < 0.0001, P1KO vs P1KO + Si-DRP1 P < 0.0001, P1KO + Si-con vs P1KO + Si-con *P* = 0.0028). The cells were stained with DAPI (blue) and antibody for Ki67 (purple) (**g**) and the percentages of Ki67-positive cells were quantified (**h**, A549 and P1KO *n* = 33 fields, P1KO + Si-con and P1KO + Si-DRP1 *n* = 31 fields; A549 vs P1KO *P* < 0.0001, A549 vs P1KO + Si-con *P* < 0.0001, P1KO vs P1KO + Si-DRP1 *P* < 0.0001, P1KO + Si-con vs P1KO + Si-con *P* < 0.0001). Bar, 25 μm. **i** The cells were cultured for 4 days and the numbers of live cells were analyzed as in Fig. 2b (*n* = 4; A549 vs P1KO *P* < 0.0001, A549 vs P1KO + Si-con *P* < 0.0001, P1KO vs P1KO + Si-DRP1 *P* < 0.0001, P1KO + Si-con vs P1KO + Si-con *P* < 0.0001). Data in **c**, **d**, **f**, **h**, and **i** represent mean ± SEM. Statistical significance was calculated using one-way ANOVA with Tukey–Kramer post-hoc analysis, **P < 0.01; ***P < 0.001. Source data are provided as a Source Data file.

To test whether PINCH-1 deficiency-induced increase of DRP1 expression was responsible for the increase of mitochondrial fragmentation (Fig. 2e–g) and inhibition of cell proliferation (Fig. 2b, d), we knocked down DRP1 from PINCH-1 KO cells (Fig. 5a, lane 4) and determined the effects. The results showed that knockdown of DRP1 effectively reversed PINCH-1 deficiency-induced increase of mitochondrial fragmentation (Fig. 5b–f) and inhibition of cell proliferation (Fig. 5g–i), suggesting that PINCH-1 regulates these processes through, at least in part, control of DRP1 level.

Consistent with a critical role of DRP1 in mediating mitochondrial division, knockdown of DRP1 from PINCH-1 expressing lung adenocarcinoma cells (Supplementary Fig. 5a, compare lane 3 with lanes 1 and 2) increased mitochondria elongation (Supplementary Fig. 5b). However, in marked contrast to the pro-cell proliferation effect of DRP1 knockdown in PINCH-1 KO lung adenocarcinoma cells (Fig. 5g–i), knockdown of DRP1 from PINCH-1 expressing lung adenocarcinoma cells reduced rather than increased cell proliferation (Supplementary Fig. 4c). Thus, the effect of DRP1 on lung adenocarcinoma cell proliferation is context or PINCH-1 dependent (i.e., DRP1 promotes cell proliferation in the presence of PINCH-1 but inhibits cell proliferation in the absence of PINCH-1).

### PINCH-1 promotes kindlin-2 interaction with PYCR1

**Fig. 6:**
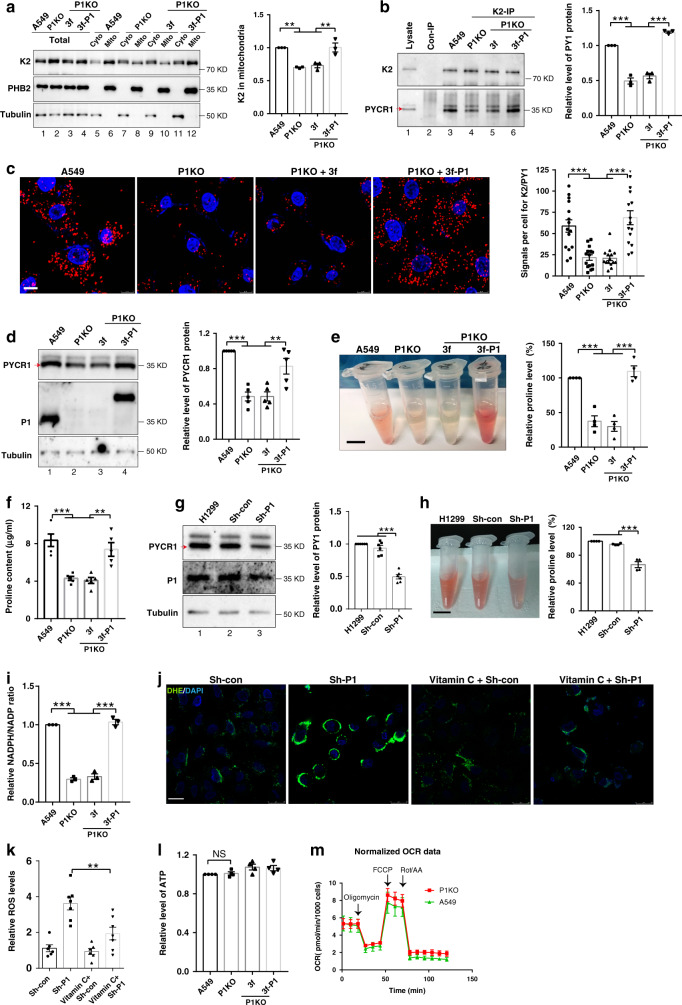
PINCH-1 promotes kindlin-2 interaction with PYCR1 and proline synthesis. **a** PINCH-1 KO A549 cells were infected with 3xFLAG- PINCH-1 (3f-P1) or 3xFLAG (3f) lentivirus. The cytosolic (Cyto) (lane 5, 7, 9, and 11), mitochondrial (Mito) (lane 6, 8, 10, and 12) and total (lane 1, 2, 3, and 4) fractions were analyzed by Western blotting. Kindlin-2 (K2) level in mitochondria was quantified (right, *n* = 3). **b** Cells were analyzed by IP and Western blotting. Lane 2, the sample was prepared as that of lane 3 except anti-kindlin-2 antibody was substituted with irrelevant mouse IgG. PYCR1 level was quantified (right, *n* = 3). **c** Cells were analyzed by PLA with kindlin-2 and PYCR1 antibodies. Bar, 10 μm. PLA dots were counted (right, *n* = 15). **d** Cells were analyzed by Western blotting. PYCR1 level in PINCH-1 KO cells was quantified and compared to that of A549 cells (right, *n* = 5). **e**, **h** The proline levels were analyzed using the absorbance method (right, *n* = 4). A representative set of samples were shown in the left. Scale bar, 1 cm. **f** The proline levels were analyzed by mass spectrometry (*n* = 5). **g** H1299 cells were infected with Sh-PINCH-1 (P1) or Sh-con lentivirus and analyzed by Western blotting. PYCR1 levels were quantified and compared to that in H1299 cells (right, *n* = 6). **i** The NADPH/NADP + ratios were analyzed. **j**, **k** ROS levels were analyzed with DHE (**j**). Scale bar, 75 μm. The mean fluorescence intensities (MFI) were calculated (k, *n* = 3). **l** ATP levels were analyzed (*n* = 4). **m** Mitochondrial respiration was analyzed using Seahorse. OCRs in wild type (green) and PINCH-1 KO (red) A549 cells before and after injection of oligomycin (1 μM), FCCP (1 μM), rotenone (Rot, 0.5 μM) and antimycin A (AA, 0.5 μM) are shown (A549 *n* = 4, KO *n* = 5). Data in **a**–**m** represent mean ± SEM. Statistical significance in **a**–**l** was calculated using one-way ANOVA with Tukey–Kramer post-hoc analysis. Statistical significance in **m** was determined using unpaired two-tail *t*-test. **P < 0.01; ***P < 0.001; NS no significance. The samples in **a**, **d**, and **g** were from same experiment and blots were processed in parallel. Source data are provided in Source Data file.

We previously showed that a fraction of kindlin-2 is translocated into mitochondria, where it forms a complex with PYCR1 and promotes proline synthesis^11^. Given the critical role of PINCH-1 in regulation of mitochondrial fragmentation (Fig. 2), we tested whether loss of PINCH-1 affects kindlin-2 mitochondrial translocation, interaction with PYCR1, and proline synthesis. Subcellular fractionation experiments showed that the level of mitochondrial kindlin-2 (Fig. 6a, compare lane 8 with lane 6) but not that of total kindlin-2 (Fig. 6a, compare lane 2 with lane 1) was reduced in PINCH-1 KO cells compared with that in control cells, suggesting that loss of PINCH-1 impairs kindlin-2 mitochondrial translocation. Furthermore, proximity ligation assay (PLA) showed that loss of PINCH-1 significantly inhibited complex formation between kindlin-2 and PYCR1 (Fig. 6c). This was confirmed by co-immunoprecipitation (IP) experiments (Fig. 6b, compare lane 4 with lane 3). Consistent with our previous study showing that kindlin-2-PYCR1 complex formation prevents PYCR1 degradation and thereby promotes proline synthesis^11^, KO of PINCH-1 from A549 cells reduced the levels of PYCR1 (Fig. 6d, compare lanes 1 and 2) and proline (Fig. 6e). Mass spectroscopic quantification confirmed that the level of proline was reduced in response to loss of PINCH-1 (Fig. 6f). Similarly, knockdown of PINCH-1 from H1299 cells also reduced the levels of PYCR1 (Fig. 6g) and proline (Fig. 6h). In addition, loss of PINCH-1 reduced the ratio of NADPH/NADP^+^ (Fig. 6i) and increased the ROS level (Fig. 6j, k). No significant changes of the levels of ATP and oxygen consumption rate (OCR) (Fig. 6l, m), however, were observed in response to loss of PINCH-1. Re-expression of 3xFLAG-tagged PINCH-1 in PINCH-1 KO cells (Fig. 6d, lane 4) restored kindlin-2 mitochondrial translocation (Fig. 6a), its complex formation with PYCR1 (Fig. 6b, c) and the levels of PYCR1 (Fig. 6d), proline (Fig. 6e, f), NADPH/NADP^+^ ratio (Fig. 6i) and ROS (Fig. 6j, k), confirming a critical role of PINCH-1 in these processes.

**Fig. 7:**
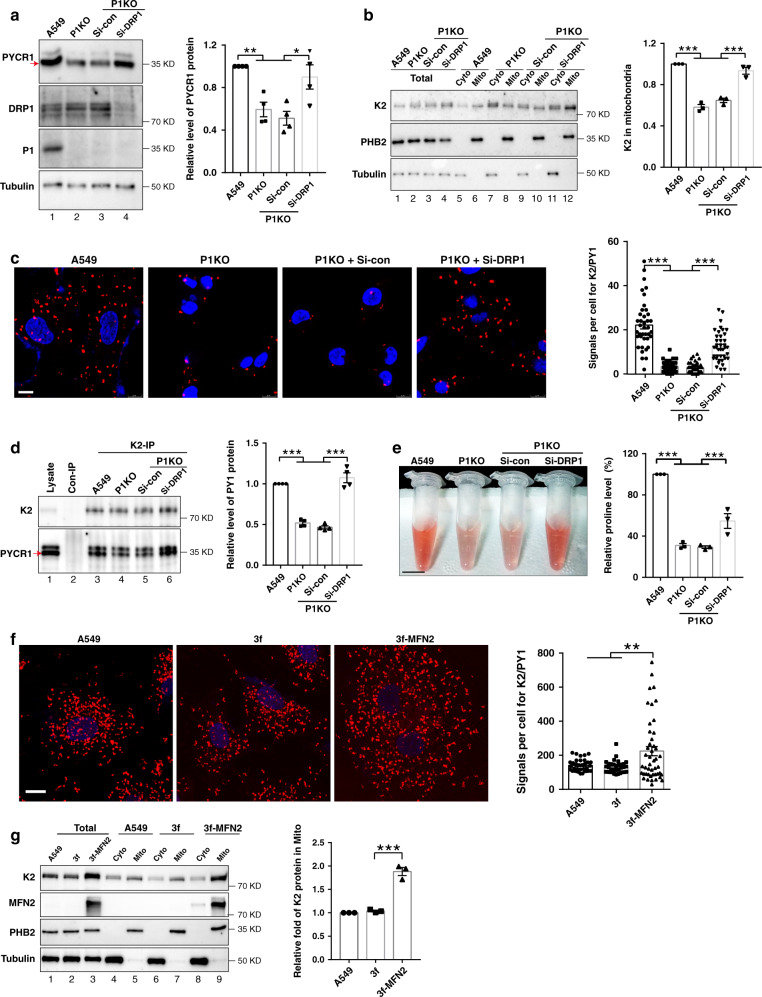
Depletion of DRP1 from PINCH-1 KO cells promotes kindlin-2-PYCR1 interaction. **a** PINCH-1 KO A549 cells were transfected with Si-DRP1 or Si-con as indicated. Three days later, cells (as indicated) were analyzed by Western blotting with antibodies recognizing PYCR1, DRP1, PINCH-1, or tubulin. PYCR1 level was quantified by densitometry (right, *n* = 4; A549 vs P1KO *P* = 0.0076, A549 vs P1KO + Si-con *P* = 0.0023, P1KO vs P1KO + Si-DRP1 *P* = 0.0419, P1KO + Si-con vs P1KO + Si-DRP1 *P* = 0.012). **b** The cytosolic (Cyto) (lane 5, 7, 9 and 11), mitochondrial (Mito) (lane 6, 8, 10, and 12) and total (lane 1, 2, 3, and 4) fractions from the cells were analyzed by Western blotting with antibodies recognizing kindlin-2 (K2), PHB2, or tubulin. Kindlin-2 level in mitochondria was quantified (right, *n* = 3; A549 vs P1KO *P* < 0.0001, A549 vs P1KO + Si-con *P* < 0.0001, P1KO vs P1KO + Si-DRP1 *P* < 0.0001, P1KO + Si-con vs P1KO + Si-DRP1 *P* < 0.0001). **c** Cells were analyzed by PLA with kindlin-2 and PYCR1 antibodies. Bar, 10 μm. The number of PLA dots per cell were counted (right, *n* = 40 cells; A549 vs P1KO *P* < 0.0001, A549 vs P1KO + Si-con *P* < 0.0001, P1KO vs P1KO + Si-DRP1 *P* < 0.0001, P1KO + Si-con vs P1KO + Si-DRP1 *P* < 0.0001). **d** Cells (as indicated) were analyzed by IP and Western blotting. Lane 2, the sample was prepared as that of lane 3 except anti-kindlin-2 antibody was substituted with irrelevant mouse IgG. PYCR1 level was quantified by densitometry (right, *n* = 4; A549 vs P1KO *P* < 0.0001, A549 vs P1KO + Si-con *P* < 0.0001, P1KO vs P1KO + Si-DRP1 *P* < 0.0001, P1KO + Si-con vs P1KO + Si-DRP1 *P* < 0.0001). **e** The proline level was analyzed using the absorbance method as described in the “Methods” (right, *n* = 3 independent experiments; A549 vs P1KO *P* < 0.0001, A549 vs P1KO + Si-con *P* < 0.0001, P1KO vs P1KO + Si-DRP1 *P* = 0.0086, P1KO + Si-con vs P1KO + Si-DRP1 *P* = 0.0058). A representative set of samples were shown in the left. Bar, 1 cm. **f** A549 cells were infected with lentiviral vector encoding 3xFLAG-tagged MFN2 (3f-MFN2) or with 3xFLAG vector (3f). Three days later, the cells were analyzed by PLA with kindlin-2 and PYCR1 antibodies. Bar, 10 μm. The number of PLA dots per cell were counted (right, A549 *n* = 34 cells, 3f *n* = 33 cells, 3f-MFN2 *n* = 46 cells; A549 vs 3f-MFN2 *P* = 0.0074, 3f vs 3f-MFN2 *P* = 0.0027). **g** The cytosolic (lane 4, 6, and 8), mitochondrial (lane 5, 7, and 9) and total (lane 1, 2, and 3) fractions from the cells were analyzed by Western blotting. The levels of kindlin-2 in the cytosolic or mitochondrial fractions were quantified by densitometry. Right panel, the ratio of the mitochondrial kindlin-2 level divided by the cytosolic kindlin-2 level in the MFN2 overexpressing cells was compared to that in the control infectants or wild type A549 cells (normalized to 1, *n* = 3; A549 vs 3f-MFN2 *P* < 0.0001, 3f vs 3f-MFN2 *P* < 0.0001). Data in **a**–**g** represent mean ± SEM. Statistical significance was calculated using one-way ANOVA with Tukey–Kramer post-hoc analysis, *P < 0.05; **P < 0.01; ***P < 0.001. The samples in **a**, **b**, **d**, and **g** were from the same experiment and the blots were processed in parallel. Source data are provided as a Source Data file.

The findings that PINCH-1 deficiency promotes mitochondrial fragmentation and concomitantly inhibits kindlin-2 mitochondrial translocation, interaction with PYCR1, and proline synthesis raised an interesting possibility that mitochondrial dynamics and kindlin-2 mitochondrial translocation and consequently its interaction with PYCR1 and proline synthesis are intrinsically linked. To test this, we knocked down DRP1, which suppressed PINCH-1 deficiency-induced mitochondrial fragmentation (Fig. 5a–f), and determined the effects on kindlin-2 mitochondrial translocation, complex formation with PYCR1 and proline synthesis. The results showed that knockdown of DRP1 from PINCH-1 KO A549 cells (Fig. 7a, lane 4) was sufficient to increase kindlin-2 mitochondrial translocation (Fig. 7b, compare lane 12 with lanes 8 and 10) and complex formation with PYCR1 (Fig. 7c, d), and reversed PINCH-1 deficiency-induced reduction of the levels of PYCR1 (Fig. 7a, compare lane 4 with lanes 2 and 3) and proline (Fig. 7e). Collectively, these results suggest that PINCH-1 regulates kindlin-2 mitochondrial translocation, its interaction with PYCR1 and the levels of PYCR1 and proline through, at least in part, control of DRP1 expression and mitochondrial fragmentation. Depletion of DRP1 from wild type A549 cells (Supplementary Fig. 4a) also increased kindlin-2 mitochondrial translocation (Supplementary Fig. 4d, compare lane 9 with lanes 5 and 7) and complex formation with PYCR1 (Supplementary Fig. 4f). However, the levels of PYCR1 (Supplementary Fig. 4d, compare lane 3 with lanes 1 and 2) and proline (Supplementary Fig. 4e) were not increased in response to depletion of DRP1 in PINCH-1 expressing wild type A549 cells. Thus, the effects of depletion of DRP1 on PYCR1 and proline synthesis are also PINCH-1 context dependent (i.e., depletion of DRP1 increased the levels of PYCR1 and proline synthesis in PINCH-1 KO but not PINCH-1 expressing cells).

To further test whether kindlin-2 mitochondrial translocation and complex formation with PYCR1 are regulated by mitochondrial fragmentation, we overexpressed MFN2, a key mediator of mitochondrial fusion, and analyzed the effects on kindlin-2 mitochondrial translocation and complex formation with PYCR1. As expected, overexpression of MFN2 reduced mitochondrial fragmentation (Supplementary Fig. 5). Furthermore, it significantly increased kindlin-2 mitochondrial translocation (Fig. 7g, compare lane 9 with lanes 5 and 7) and complex formation with PYCR1 (Fig. 7f), confirming that these processes are controlled by mitochondrial fragmentation.

### PYCR1 mediates the effect of PINCH-1 on proline synthesis

**Fig. 8:**
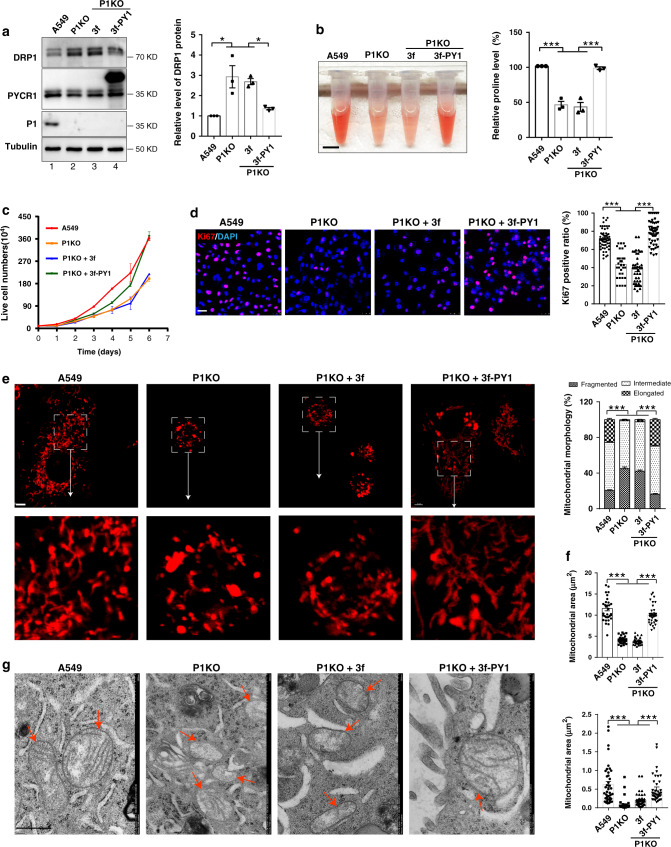
Overexpression of PYCR1 in PINCH-1 KO cells restores proline synthesis. **a** PINCH-1 KO A549 cells were infected with 3xFLAG-PYCR1 (3f-PY1) or 3xFLAG (3f) lentivirus. The cells were analyzed by Western blotting with antibodies as indicated. DRP1 level was quantified (right, *n* = 3; A549 vs P1KO *P* = 0.006, A549 vs P1KO + 3f *P* = 0.013, P1KO vs P1KO + 3f-PY1 *P* = 0.019, P1KO + 3f vs P1KO + 3f-P1 *P* = 0.0434). The samples were from same experiment and blots were processed in parallel. **b** The proline level was analyzed using the absorbance method (right, *n* = 3 independent experiments; A549 vs P1KO *P* < 0.0001, A549 vs P1KO + 3f *P* < 0.0001, P1KO vs P1KO + 3f-PY1 *P* < 0.0001, P1KO + 3f vs P1KO + 3f-P1 *P* < 0.0001). A representative set of samples were shown in the left. Bar, 1 cm. **c** The numbers of live cells (A549 cells, red line; P1 KO cells, orange line; 3f lentiviral vector infected P1 KO cells, blue line; 3f-PY1 lentiviral vector infected P1 KO cells, green line) were analyzed as in Fig. 2b (*n* = 3). **d** Cells were stained with DAPI (blue) and Ki67 antibody (purple). Bar, 25 μm. The percentages of Ki67 positive cells were quantified (right, A549 *n* = 56 fields, P1KO *n* = 33 fields, P1KO + 3f *n* = 42 fields, P1KO + 3f-PY1 *n* = 51 fields; A549 vs P1KO *P* < 0.0001, A549 vs P1KO + 3f *P* < 0.0001, P1KO vs P1KO + 3f-PY1 *P* < 0.0001, P1KO + 3f vs P1KO + 3f-P1 *P* < 0.0001). **e** Mitochondria were stained with MitoTracker Red CMXRos and mitochondria with different morphologies were quantified (right, A549 and P1KO *n* = 50 cells, P1KO + 3f *n* = 40 cells, P1KO + 3f-PY1 *n* = 37 cells; A549 vs P1KO *P* < 0.0001, A549 vs P1KO + 3f *P* < 0.0001, P1KO vs P1KO + 3f-PY1 *P* < 0.0001, P1KO + 3f vs P1KO + 3f-P1 *P* < 0.0001). Bar, 5 μm. **f** Mitochondrial areas in z-stack images were quantified (A549 *n* = 33 cells, P1KO *n* = 35 cells, P1KO + 3f *n* = 36 cells, P1KO + 3f-PY1 *n* = 37 cells; A549 vs P1KO *P* < 0.0001, A549 vs P1KO + 3f *P* < 0.0001, P1KO vs P1KO + 3f-PY1 *P* < 0.0001, P1KO + 3f vs P1KO + 3f-P1 *P* < 0.0001). **g** Mitochondria (red arrows) were observed under TEM (left panels, bar, 500 nm) and mitochondria areas were quantified (right panel, *n* = 40 mitochondria; A549 vs P1KO *P* < 0.0001, A549 vs P1KO + 3f *P* < 0.0001, P1KO vs P1KO + 3f-PY1 *P* < 0.0001, P1KO + 3f vs P1KO + 3f-P1 *P* = 0.001). Data in **a**–**g** represent mean ± SEM. Statistical significance was calculated using one-way ANOVA with Tukey–Kramer post-hoc analysis, *P < 0.05; ***P < 0.001. Source data are provided in Source Data file.

We next tested whether the reduction of PYCR1 level (Fig. 6a, compare lanes 1 and 2) was involved in the inhibition of proline synthesis and cell proliferation induced by the loss of PINCH-1 (Figs. 2b, d and 6e, f). To do this, we overexpressed PYCR1 in PINCH-1 KO cells (Fig. 8a, lane 4). The results showed that this reversed PINCH-1 deficiency-induced inhibition of proline synthesis (Fig. 8b) and cell proliferation (Fig. 8c, d). Thus, PINCH-1 deficiency-induced reduction of PYCR1 level was critical for inhibition of proline synthesis and cell proliferation found in PINCH-1 KO cells. Interestingly, overexpression of PYCR1 in PINCH-1 KO cells reversed the increase of DRP1 expression (Fig. 8a) and mitochondrial fragmentation (Fig. 8e–g), suggesting that PYCR1 functions in not only control of proline synthesis but also regulation of DRP1 expression and mitochondrial fragmentation.

### Ablation of PINCH-1 inhibits tumor growth in vivo

**Fig. 9:**
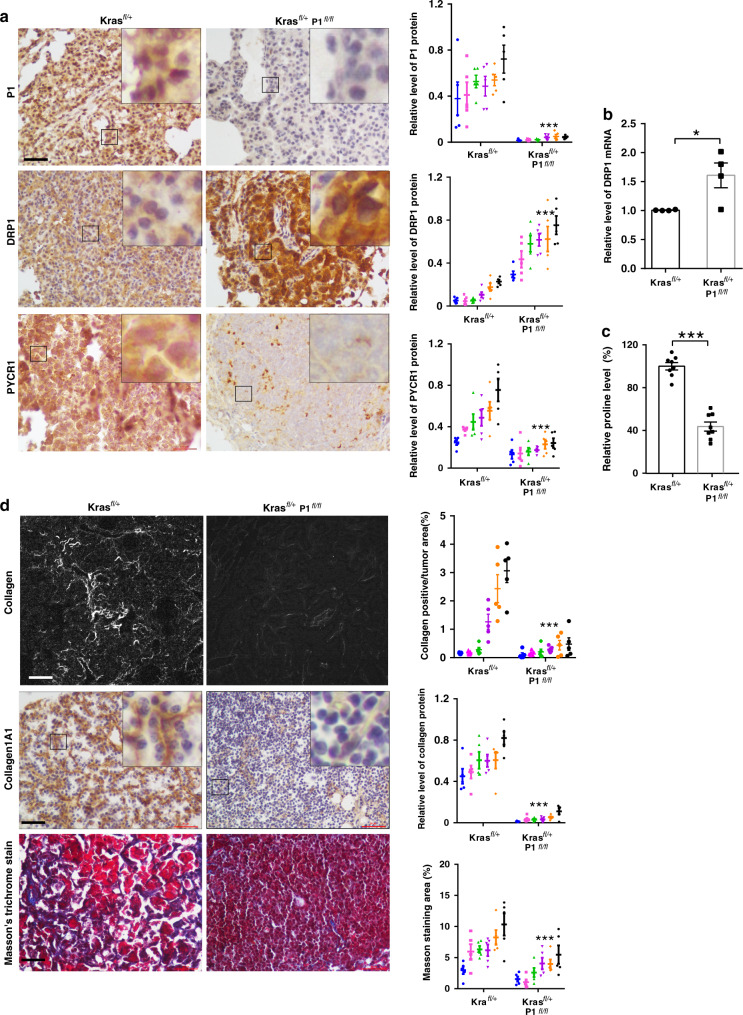
Ablation of PINCH-1 reduces proline and collagen matrix synthesis in vivo. The lung of the mice was administrated with Ad-Cre and analyzed 16 weeks later. **a** Sections of the lung tissues from the mice (as specified in the figure) were analyzed by immunostaining with antibodies for PINCH-1 (P1)(top), DRP1 (middle), or PYCR1 (bottom). Bar, 20 μm. The boxed areas in the IHC staining were enlarged and shown in the upper right corner. Right panels, the mean intensities of PINCH-1, DRP1, and PYCR1 staining in the Kras^LSL−G12D/+^; PINCH-1(P1)^fl/fl^ (Kras ^fl/+^; P1^fl/fl^) group were quantified and compared to those of the Kras^fl/+^ group (normalized to 1; *n* ≥ 30 fields from 6 mice for each group; different mice in each group were coded with different colors; *P* < 0.0001). **b** The DRP1 mRNA levels from the lung tissues (as indicated) were analyzed by RT-PCR (*n* = 4; *P* = 0.0307). **c** The proline levels in the lung tissues were analyzed as described in the Methods (*n* = 8 mice; *P* < 0.0001). **d** Collagen matrix was analyzed by SHG with multiphoton microscopy (top; bar, 100μm), immunostaining with antibody for collagen1A1 (middle; bar, 20 μm), or Masson’s trichrome staining (bottom; bar, 20 μm). The boxed areas in the IHC staining were enlarged and shown in the upper right corner. Bottom panels, for each method (as indicated in the figure) the mean intensities of collagen matrix in the Kras^fl/+^; P1^fl/fl^ group were quantified and compared to those of the Kras^fl/+^ group (normalized to 1; *n* = 30 fields from 6 mice for each group; different mice in each group were coded with different colors; *P* < 0.0001). Data in **a**–**d** represent mean ± SEM. Statistical significance was calculated using two-tailed unpaired Student’s *t*-test, *P < 0.05; ***P < 0.001. Source data are provided as a Source Data file.

**Fig. 10:**
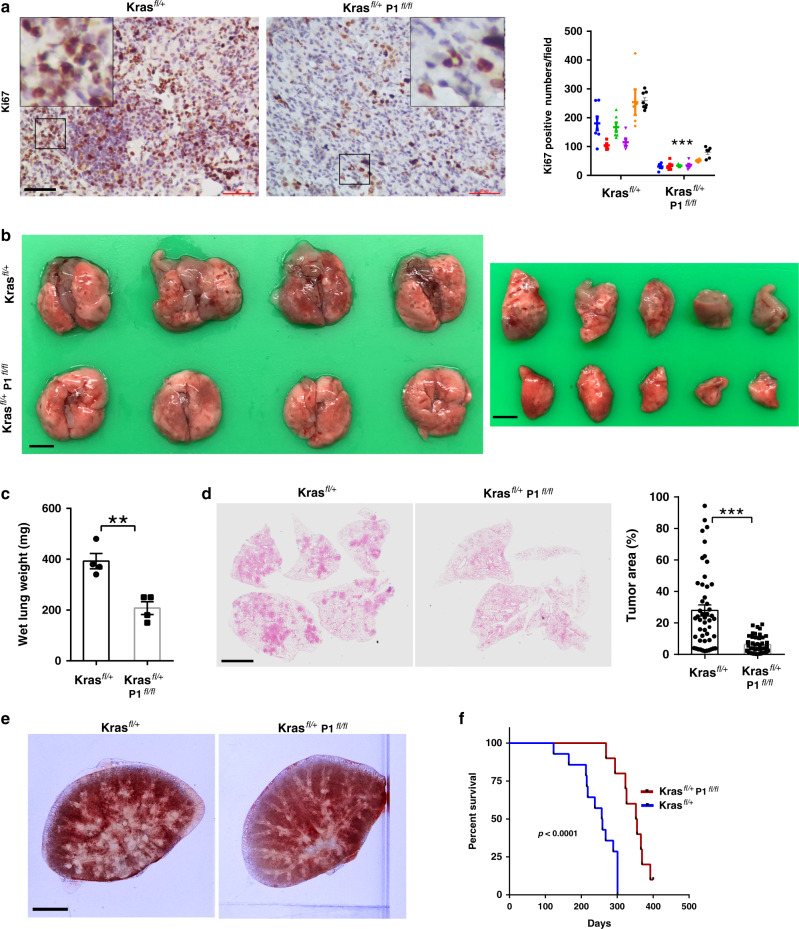
Ablation of PINCH-1 inhibits lung adenocarcinoma growth in vivo. The lung of the mice (as indicated in the figure) was administrated with Ad-Cre and analyzed 16 weeks later. **a** Sections of the lung tissues from the mice (as specified in the figure) were analyzed by immunostaining with antibodies for Ki67. Bar, 20 μm. Right panels, the percentages of Ki67 positive cells in the lung tissues derived from the Kras^LSL−G12D/+^; PINCH-1^fl/fl^ (Kras^fl/+^; P1^fl/fl^) group were quantified and compared to those of the Kras^fl/+^ group (Kras^fl/+^ group *n* = 36 fields from 6 mice, Kras^fl/+^; P1^fl/fl^ group *n* = 35 fields from 6 mice, different mice in each group were coded with different colors; *P* < 0.0001). **b** The gross morphology of the lung was observed. Scale bars = 1 cm. **c** The lung tissues from the mice were weighed (*n* = 4; *P* = 0.0034). **d** Sections of the lung tissues from the mice were analyzed by H&E staining. Scale bar = 200 μm. Tumor area was quantified as described in the Methods (*n* ≥ 30 fields from 4 mice; *P* < 0.0001). **e** The lung was pressed onto two slides and observed under microscopy for analyses of lung tumors. Scale bar = 0.5 cm. **f** Kaplan–Meier survival curve of Kras^fl/+^ mice (*n* = 14) vs. Kras^fl/+^; P1^fl/fl^ mice (*n* = 11) for up to 400 days post-administration of Ad-Cre. Kaplan–Meir survival analysis was determined by log-rank test. Data in **a**, **c**, **d** present mean ± SEM. Statistical significance was calculated using two-tailed unpaired Student’s *t*-test, **P < 0.01; ***P < 0.001. Source data are provided as a Source Data file.

To test whether PINCH-1 regulates DRP1, PYCR1, proline synthesis, and tumor growth in vivo, we ablated PINCH-1 from Kras^G12D^-induced lung adenocarcinoma in mice, which normally expressed a high level of PINCH-1 (Fig. 1b). To do this, we crossed PINCH-1^fl/fl^ mice with Kras^LSL-G12D/+^(Kras^fl/+^) mice and obtained Kras^LSL-G12D/+^**;** PINCH-1^fl/fl^ (Kras^fl/+^; P1^fl/fl^) and Kras^fl/+^ mice, respectively. Adenovirus encoding Cre (Ad-Cre) was administrated into the lung to induce expression of Kras^G12D^ and inactivation of *PINCH-1* gene. Consistent with the results from cultured lung adenocarcinoma cells (Figs. 4 and 6), ablation of PINCH-1 from lung adenocarcinoma significantly increased the protein (Fig. 9a) and mRNA (Fig. 9b) levels of DRP1 and reduced the levels of PYCR1 (Fig. 9a) and proline (Fig. 9c) in vivo. Furthermore, the level of collagen matrix was also significantly reduced (Fig. 9d). Similar to what we found in culture (Fig. 2), ablation of PINCH-1 significantly reduced cell proliferation (Fig. 10a) in vivo. Importantly, while expression of Kras^G12D^ markedly induced lung tumor formation in Kras^LSL-G12D/+^ mice (Fig. 10b), the tumors formed in Kras^LSL-G12D/+^; PINCH-1^fl/fl^ mice administrated with Ad-Cre were significantly smaller compared to those in Kras^LSL-G12D/+^ mice administrated with Ad-Cre (Fig. 10b–e). Consistent with this, ablation of PINCH-1 significantly reduced the mortality rate of the mice with lung adenocarcinoma (Fig. 10f).

## Discussion

Reprograming of proline metabolism is critically involved in tumor growth. Identification of signaling pathways that control proline synthesis, therefore, is important for elucidation of the mechanisms governing tumor growth and could potentially lead to development of therapeutic approaches to alleviate tumor growth. Using tissues from human patients with lung adenocarcinoma (Fig. 1a–c) as well as those from Kras^G12D^-induced lung adenocarcinoma in mice (Fig. 1e), we show that the level of PINCH-1 is significantly increased in lung adenocarcinoma, which is correlated with poorer human patient survival (Fig. 1d). Human lung adenocarcinoma cells are capable of synthesizing a large amount of proline (Fig. 6f). KO of PINCH-1 from human lung adenocarcinoma cells in culture (Fig. 6a, e) as well as lung adenocarcinoma in mouse (Fig. 9a, c) reduced the levels of PYCR1 and proline. Furthermore, the level of collagen matrix (Fig. 9d), of which 25% amino acids were derived from proline, as well as cell proliferation (Figs. 2b, d, 3b–d, 5g–i, 8c, d) and tumor growth (Fig. 10a–e) were markedly reduced in response to loss of PINCH-1, resulting in significant improvement of the mortality rate of mice bearing lung adenocarcinoma (Fig. 10f). These findings suggest that elevated level of PINCH-1 and consequently those of PYCR1 and proline are critical for promotion of lung adenocarcinoma cell proliferation and tumor growth.

How does PINCH-1 regulate PYCR1 and proline synthesis? Unlike kindlin-2^11^, immunofluorescent staining experiments failed to detect PINCH-1 or its associated proteins ILK and α-parvin in the mitochondria (Supplementary Fig. 2). Thus, although we cannot completely rule out the possibility that a small amount of PINCH-1 that is beyond the detection limit of immunofluorescent staining localizes to the mitochondria and directly contributes to the regulation of PYCR1 and proline synthesis, several lines of evidence suggest that PINCH-1 regulates mitochondrial fragmentation, PYCR1, and proline synthesis indirectly. First, loss of PINCH-1 significantly increased DRP1 mRNA level (Fig. 4d, e), suggesting that PINCH-1 may regulate DRP1 through, at least in part, control of *DRP1* gene transcription. Second, KO of PINCH-1 enhanced mitochondrial fragmentation (Fig. 2e–g), which was reversed by the depletion of DRP1 (Fig. 5b–f), suggesting that PINCH-1 influences mitochondrial fragmentation through regulation of DRP1 expression. Third, in previous studies we have shown that a fraction of kindlin-2 is translocated from cytosol to mitochondria where it forms a complex with PYCR1, prevents proteolytic degradation of PYCR1 and thereby promotes proline synthesis^11^. The cellular mechanism that controls kindlin-2 mitochondrial translocation, complex formation with PYCR1 and consequently proline synthesis, however, was not known. In this study, we show that KO of PINCH-1, which increased DRP1 expression and mitochondrial fragmentation (Figs. 2–4), markedly reduced kindlin-2 mitochondrial translocation, complex formation with PYCR1 and proline synthesis (Fig. 6). Furthermore, depletion DRP1, which reversed mitochondrial fragmentation (Fig. 5b–f), restored kindlin-2 mitochondrial translocation, complex formation with PYCR1 and proline synthesis (Fig. 7b–e). As expected, increase of PYCR1 expression in PINCH-1 KO cells restored proline synthesis (Fig. 8b) and cell proliferation (Fig. 8c, d). Collectively, these findings suggest that PINCH-1 regulates PYCR1 and proline synthesis through, at least in part, control of DRP1 expression and consequently mitochondrial fragmentation, kindlin-2 mitochondrial translocation and complex formation with PYCR1. Consistent with an essential role of kindlin-2 in PINCH-1-mediated regulation of PYCR1 and proline synthesis, overexpression of PINCH-1 was unable to increase the levels of PYCR1 and proline in the absence of kindlin-2 (Supplementary Fig. 6). Interestingly, increase of PYCR1 expression reversed the PINCH-1 deficiency-induced increase of DRP1 expression (Fig. 8a) and mitochondrial fragmentation (Fig. 8e–g), suggesting that there is a feedback system through which PYCR1 or proline synthesis regulates DRP1 expression and mitochondrial dynamics. In other words, DRP1 and mitochondrial dynamics not only exert a strong influence on PYCR1 and proline synthesis but they themselves are also regulated by PYCR1 or proline synthesis. This feedback system may help cells to respond quickly the need for increase proline synthesis in fasting proliferating cells such as cancer cells. In this regard, it is worth noting that deprivation of cellular nutrition is known to have a strong impact on mitochondrial dynamics^14,15,47–50^. It will be interesting to determine in future studies whether PYCR1 regulates DRP1 and mitochondrial dynamics directly or indirectly through control of proline synthesis and the specific signaling pathways that are involved.

In addition to showing that PINCH-1 regulates proline synthesis, cell proliferation, collagen matrix deposition, and tumor growth and revealing a signaling axis consisting of PINCH and DRP1 that functions in regulation of these processes, the findings presented in this paper suggest that the function of DRP1 in regulation of proline synthesis and cell proliferation is context (i.e., PINCH-1) dependent. Consistent with previous studies^51^, depletion of DRP1 from lung adenocarcinoma cells, which express a high level of PINCH-1 (Fig. 1), inhibited cell proliferation (Supplementary Fig. 4c). However, in contrast to the pro-proliferation role of DRP1 in PINCH-1 expressing lung adenocarcinoma cells, knockdown of DRP1 from PINCH-1 deficient lung adenocarcinoma cells (Fig. 5a), which expressed an elevated level of DRP1 (Fig. 4a) and exhibited excessive mitochondrial fragmentation (Fig. 2e–g), increased cell proliferation (Fig. 5g–5i). Of note, knockdown of DRP1 in PINCH-1 KO lung adenocarcinoma cells (Fig. 7a, e), but not that in PINCH-1 expressing wild type lung adenocarcinoma cells (Supplementary Fig. 4d, e), significantly increased the level of PYCR1 and proline synthesis. The difference in the effect of depletion of DRP1 on the level of PYCR1 (and consequently proline synthesis) probably reflects the different PYCR1 degradation rates in the PINCH-1 KO and PINCH-1 expressing cells (i.e., loss of PINCH-1 KO significantly increases PYCR1 degradation). This is consistent with the findings that degradation of PYCR1 is markedly increased in response to inhibition of kindlin-2 mitochondrial translocation and complex formation with PYCR1^11^ and loss of PINCH-1 inhibited kindlin-2 mitochondrial translocation and complex formation with PYCR1 (Fig. 6). Thus, excessive mitochondrial fragmentation such as that found in PINCH-1 deficient lung adenocarcinoma cancer cells is detrimental to cell proliferation, which probably due at least in part to the inhibitory role of mitochondrial fragmentation on proline synthesis as shown in this study and the requirement of an adequate supply of proline for optimal cell proliferation as demonstrated by previous studies^1,2,10^.

While it is clear that PINCH-1 plays an important role in regulation of *DRP1* expression, the downstream transcription factors or cofactors mediating the effect of PINCH-1 remain to be determined. Previous studies have implicated a role of hypoxia-inducible factor-1α (HIF-1α)^52^ and peroxisome proliferator-activated receptor-gamma coactivator 1 (PGC-1) α and β^53^ in regulation of *DRP1* expression. It will be interesting to test in future studies whether these transcription factor and cofactors are involved in PINCH-1 mediated regulation of *DRP1* expression. In this regard, it is worth noting that depletion of ILK, like that of PINCH-1, reduced the DRP1 level (Fig. 4f), suggesting that PINCH-1 works in concert with ILK in regulation of *DRP1* expression. By contrast, KO of kindlin-2 failed to alter the level of DRP1 (Fig. 4i), suggesting that DRP1 is a selective downstream effector of PINCH-1 and ILK. Nevertheless, our preliminary studies suggest that KO of kindlin-2 did increase mitochondrial fragmentation (Supplementary Fig. 7a, c), indicating that kindlin-2 may also regulate mitochondrial dynamics, albeit through a signaling mechanism that is different from that of PINCH-1 and ILK. Interestingly, re-expression of the ILK-binding defective L353A/L357A (LLAA) kindlin-2 mutant^54,55^, like that of wild type kindlin-2, effectively reversed the increase of mitochondrial fragmentation and inhibition of proline synthesis induced by the loss of kindlin-2 (Supplementary Fig. 7b, c, f). By contrast, re-expression of the integrin-binding defective W619Q kindlin-2 mutant^56^, unlike that of wild type kindlin-2 or the LLAA mutant, failed to reverse the increase of mitochondrial fragmentation and inhibition of proline synthesis induced by the loss of kindlin-2 (Supplementary Fig. 7a, c, e). Consistent with a critical role of the complex formation between kindlin-2 and PYCR1 in regulation PYCR1 level and proline synthesis, the ability of the W619Q mutant to form a complex with PYCR1 was diminished compared to that of wild type kindlin-2 and the LLAA mutant (Supplementary Fig. 7d). Clearly, future studies are required to further delineate the signaling mechanism through which kindlin-2 regulates mitochondrial dynamics.

## Methods

### Mice

PINCH-1^*fl/fl*^ transgenic mice were generated in a previous study^57,58^. Kras^LSL-G12D/+^ mice were bought from the Jackson Laboratory. All mouse work was performed with the approval of the Institutional Animal Care and Use Committee, Southern University of Science and Technology.

### Mouse genotyping and recombinant allele detection

Genotyping of LSL-Kras^*G12D*^ and floxed PINCH-1 alleles was performed by PCR using the following oligonucleotide primers^11,57,58^: LSL-KrasG12D wild forward primer 5′ GTCGACAAGCTCATGCGGG 3′; LSL-KrasG12D common reverse primer 5′ CGCAGACTGTAGAGCAGCG 3′; LSL-KrasG12D mutant forward primer 5′ CCATGGCTTGAGTAAGTCTGC 3′; PINCH1 forward primer 5′ CCCAGAAGGACTCTTTTATGAG 3′; PINCH1 reverse primer 5′ CTTGGAGAAGAAGTACTCAGGT 3′, The recombinant alleles were analyzed using genomic DNA extracted from the tips of mouse tails.

### Ad-Cre infection of mouse lung

To activate Kras^*G12D*^ in the lung, intranasal administration of Ad-Cre was performed^11,59^. To do this, age-matched mice (6–8 weeks old) of both sexes were anesthetized by intra-peritoneal injection of 20 mg ml^−1^ avertin at room temperature. Adenovirus in a calcium phosphate coprecipitate (Ad-Cre:CaPi coprecipitates) were prepared by mixing Ad-Cre (purchased from Hanbio, Shanghai, China) at the dose of 3 × 10^7^ pfu in a total volume of 90 μl and CaCl2 (at a final concentration of 10 mM CaCl2). The Ad-Cre:CaPi coprecipitates were loaded in a pipet tip and administered nasally using two 45 μl instillations with a 3 min interval.

### Quantification of tumor areas

H&E-stained slides were scanned at ×2.5 objective magnification with a digital camera (DS-Fi1c; Nikon) and NIS-Elements F Ver4.30.01 image analysis software (Nikon). Lung tumor areas were quantified using Image J software (version 2.0.0-rc-69/1.52p, NIH, Bethesda, MD, USA) in manual measurement mode.

### Immunohistochemical staining

Lung organs from mice at 16 weeks after Ad-Cre infection were isolated, fixed in 10% formalin, and embedded in paraffin^60^. Sections (5 μm thick) were cut for H&E staining and examined under a microscope. Immunohistochemistry was performed using the MaxVision^TM^ HRP-Polymer anti-Rabbit IHC Kit (MXB biotechnologies) with rabbit antibodies against PINCH-1 (Abcam, ab108609,1:800), PYCR1 (Proteintech,13108-1-AP,1:800), DRP1 (Proteintech,12957-1-AP,1:800), Ki67 (CST,12202P, 1:1000) or collagen 1A1 (Novus,NB600-408). Sections were developed with DAB and counterstained with hematoxylin. The mean optical density (MOD) of PINCH-1, PYCR1, DRP1 or collagen1A1 immunostaining was quantified using Image-Pro Plus software version 6 (Media Cybernetics, Silver Spring, MD). The region of interest was selected and then the integrated optical density (IOD) of the selected area (IOD per unit area) was measured using the software. The IOD per unit area represents the MOD of the PINCH-1, PYCR1, DRP1, or collagen1A1 staining within the tumor tissues. The highest MOD of the PINCH-1, PYCR1, DRP1, or collagen1A1 staining was normalized to 1. For quantification of Ki67 or cleaved caspase-3 staining, the numbers of Ki67 or cleaved caspase-3 positive cells in each field were manually counted. For each experimental group, six sections of lung tissues from 6 individual mice were selected and more than 5 fields for each section were analyzed.

Lung adenocarcinoma tissue microarrays were purchased from the National Engineering Center for BioChips in Shanghai, China. A more complete description of the human specimens is included in Supplementary Data 1. For each tissue section of the microarrays, ImageJ (version 2.0.0-rc-69/1.52p, NIH, Bethesda, MD, USA) was used to quantify both the DAB stained area and total tumor area. The ratio of the two parameters (DAB stained area divided by total tumor area) represents the relative expression level of PINCH-1 in the lung tissues. Although it was difficult to determine PINCH-1 subcellular localization in the IHC staining of human and mouse lung cancer tissues, comparison of PINCH-1 staining between normal and cancerous lung tissues showed that PINCH-1 level was markedly higher in human and mouse lung cancer tissues than that in normal human and mouse lung tissues (Fig. 1c, e).

### Cell culture, viral vector generation, and infection

Human A549 (ATCC^®^ CCL-185™) and NCI-H1299 (H1299, ATCC^®^ CRL-5803™) lung adenocarcinoma cells were cultured in DMEM supplemented with 10% FBS (Gibco-Invitrogen), 50 U ml^−1^ penicillin, and streptomycin at 37 °C in 5% CO_2_. Cell line authentication and mycoplasma testing were performed by ATCC. The pLKO.1, psPAX2, and pMD2.G vectors were from Addgene. pLKO.1 vectors expressing short hairpin RNAs (shRNAs) targeting human PINCH-1 or scrambled shRNA (sh-con) sequence were generated using the following sequences: Sh-PINCH-1: 5′-AAGGTGATGTGGTCTCTGCTC-3′; Sh-ILK: 5′- CGACCCAAATTTGACATGATT-3′; Sh-con: 5′-ACGCATGCATGCTTGCTTT-3′. To generate lentiviruses encoding the above shRNAs, 293T cells were co-transfected with pLKO.1 encoding various shRNAs, psPAX2, and pMD2. G. The expression vectors encoding human PINCH-1, MFN2, and kindlin-2 W619Q mutant were generated by cloning the corresponding cDNA sequences into the pLVX vector. To generate lentiviruses, vectors encoding human pLVX-PINCH-1, pLVX-MFN2, pLVX-kindlin-2 W619Q or ILK-binding defective kindlin-2 L353A/L357A (LLAA) mutant^54,55^ were co-transfected with psPAX2 and pMD2.G into 293T cells. After the cells were incubated at 37 °C, 5% CO_2_ for 24–48 h, the media contained lentiviral particles were harvested. For lentiviral infection, A549 cells were cultured in basal growth medium until 70% confluence and then replaced with fresh medium containing lentivirus (as specified in each experiment) at a multiplicity of infection (MOI) of 100 for 16 h. Lentiviral infections were carried out in the presence of 8 μg ml^−1^ polybrene.

### Generation of PINCH-1 KO A549 cells

Kindlin-2 KO A549 cells were generated in a previous study^11^. PINCH-1 KO A549 cells were generated using CRISPR/Cas9-mediated gene editing. A guide RNA (gRNA) oligoes designed to target the sequence of 5 ′- CATGAATAACAGCTGGCATC-3′ located at the exon 4 of *PINCH-1*, were cloned into pSpCas9(BB)−2A-GFP (PX458 containing cas9, Addgene plasmid # 48138) via BbsI sites and transfected into A549 cells. Singular GFP-positive cells were sorted into each wells of 96-well plate by FACS sorter (BD FACS AriaTMIII) for further propagation. Individual PINCH-1 KO colonies were examined and confirmed by DNA sequencing and Western blotting.

### RNA interference

Small interfering RNA (siRNA) directed against human DRP1 was synthesized by Invitrogen. The sense sequences of siRNAs were as followed: DRP1 siRNA: 5′- AAGCAGAAGAAUGGGGUAAAUTT −3′; control siRNA: 5′-UUCUCCGAACGUGUCACGUTT-3′. Transfection of siRNAs into A549 cells was carried out using Lipofectamine^®^ RNAiMAX Reagent (Life Technologies). Cells were transfected with 25 pmol siRNA per well in six-well plate (1 × 10^5^ cells per mL in one well).

### Trypan blue exclusion assay

Cells were seeded (10 × 10^4^ cells per ml in DMEM) in six-well tissue culture plates. The plates were incubated at 37 °C in the presence of 5% CO_2_ for different periods of time as specified in each experiment. At the end of indicated culture time, cell culture media were aspirated, and the plates were washed with PBS twice and then replenished with 1 mL of 0.05% (2 mg mL^−1^) trypsin-ethylenediaminetetraacetic acid (EDTA) solution. After incubation at 37 °C for 2 min, the cells were harvested by centrifugation at 1200 rpm for 5 min. The cell suspension (10 µL) was mixed with 10 µL of 0.4% trypan blue solution (Sigma-Aldrich, T8154, UK) and the dye-excluding viable cells were counted using a Count*star instrument.

### Cell-ECM adhesion assay

The wild type or PINCH-1 KO A549 cells were seeded in wells (60,000 cells per well) of 48-well cell plates that were pre-coated with collagen Ɩ (20ug ml^−1^, Corning, #354249), laminin (20ug ml^−1^, Sigma-Aldrich, L2020), fibronectin (20ugml^−1^, EMD Millipore, FC010) or Bovine Serum Albumin (BSA, 10 μg ml^−1^, Sigma-Aldrich, A1933) as a negative control. The cells were incubated at 37 °C in the presence of 5% CO_2_ for 1 h. At the end of incubation, the wells were washed with PBS. After washing, the cells attached to the wells were stained with 1% crystal violet. The dye was dissolved with dimethyl sulfoxide overnight at room temperature, transferred to wells of a 96-well cell plate, and the absorbance at 595 nm was quantified with a microplate reader (Biotek, Epoch2, USA).

### Cell spreading assay

The wild type or PINCH-1 KO A549 cells were allowed to adhere and spread on glass coverslips precoated with collagen Ɩ (20ug ml^−1^, Corning, #354249), laminin (20ug ml^−1^, Sigma-Aldrich, L2020) or fibronectin (20 ug ml^−1^, EMD Millipore, FC010) in a cell culture incubator at 37 °C in the presence of 5% CO_2_ for 2 h. The coverslips were washed three times with PBS, and the adhered cells were fixed with 4% paraformaldehyde and stained with Alexa Fluor–phalloidin (Life Technologies). The area of cell spreading was quantified using Image J software (version 2.0.0-rc-69/1.52p, NIH, Bethesda, MD, USA).

### Mitochondrial DNA (mtDNA) quantitation

The relative level of mtDNA (the amount of mtDNA relative to that of nuclear DNA) in the wild type or PINCH-1 KO A549 cells was quantified by RT-PCR based on the difference in the threshold amplification between mtDNA and nuclear DNA (the ΔΔC(t) method)^61^. The following primers were used in the RT-PCR to detect mtDNA and nuclear DNA, respectively:

mtDNA forward primer, CCTATCACCCTTGCCATCAT;

mtDNA reverse primer, GAGGCTGTTGCTTGTGTGAC;

nuclear DNA (*Pecam* gene) forward primer, ATGGAAAGCCTGCCATCATG;

nuclear DNA (*Pecam* gene) reverse primer, TCCTTGTTGTTCAGCATCAC.

### RT-PCR analysis

cDNA was synthesized from 10 ng total RNA using the ReverTra^®^ Ace qPCR RT Master Mix (Toyobo Life Science, Osaka, Japan) according to the manufacturer’s protocol. RT-PCR was performed using SYBR^®^ Premix Ex Taq™ II with LightCycler^®^ 480 Instrument II System. For quantifying DRP1 mRNAs, the level of glyceraldehyde-3-phosphate dehydrogenase (GAPDH) mRNA was used as an internal control and quantified in parallel with DRP1 mRNAs. Normalization and fold changes were calculated using the ∆∆Ct method. The sequences of the primers are shown in Supplementary Table 1.

### Subcellular fractions and Western blotting

For preparation of total cell lysates, cells were lysed in 1% SDS lysis buffer (25 mMTris-HCl (Ph6.8), 50 mM DTT, 10% glycerin, 2.5% sucrose). Cytosolic and mitochondrial protein fractions were prepared using a cell mitochondria isolation kit (Beyotime, C3601). Equal amounts (5–40 μg per lane) of cell proteins were separated on 10% polyacrylamide gel and transferred onto nitrocellulose membranes. Membranes were then blocked for 1 h at room temperature in Tris-buffered saline (50 mM Tris-HCl, 150 mM NaCl, pH 8.0) containing 0.1% Tween 20 and 5% non-fat powdered milk, followed by incubation at 4 °C overnight with rabbit antibodies recognizing PINCH-1 (Proteintech,20772-1-AP,1:1000), ILK(CST, 3856S,1:1000), α-Parvin(CST, 8190S,1:1000), PYCR1 (Proteintech,13108-1-AP,1:1000), kindlin-2 (Proteintech,11453-1-AP,1:1000), prohibitin-2 (PHB2, CST,14085S,1:1000), DRP1 (Proteintech,12957-1-AP,1:1000), OPA1 (Proteintech,27733-1-AP,1:1000), MFN1 (Proteintech,13798-1-AP,1:1000), MFN2 (Proteintech,12186-1-AP,1:1000), MFF (Proteintech,17090-1-AP,1:1000) or FIS1(Proteintech,10956-1-AP,1:1000), or a HRP-conjugated mouse anti-tubulin antibody (Proteintech,HRP-66031,1:5000) and then washed. The blots incubated with rabbit primary antibodies were incubated with HRP-conjugated secondary anti-rabbit IgG antibody (Jackson ImmunoResearch, #711-005-152,1:10000). The blots were developed using an enhanced chemiluminescence kit (ECL Kit, Bio-Rad) or the Ultra ECL Western Blotting Detection Reagent (4A Biotech Co., Ltd., 4AW011) and then exposed using an automatic digital gel image analysis system (Tanon, 6100B). Quantitation of band integrated density was performed with Image J (version 2.0.0-rc-69/1.52p, NIH, Bethesda, MD, USA). All experiments were repeated at least three times. Western blots were cropped to optimize clarity and presentation. Uncropped and unprocessed scans of the blots are shown in the Source Data file.

### Immunofluorescence

Cells (as specified in each experiment) were seeded on fibronectin-coated coverslips in 24 well plates (2 × 10^4^ cells per well) and cultured overnight. Cells were fixed with 4% paraformaldehyde (PFA), washed 3 times with PBS, immersed in 0.1% Triton X-100 in PBS for 10 min at room temperature, and then washed 3 times with PBS again and incubated with rabbit antibodies recognizing PINCH-1 (Abcam, ab108609,1:100), ILK(CST, 3856 S,1:200), α-Parvin (CST, 8190S,1:200), Ki67 (CST,12202P, 1:1000) or a mouse antibody recognizing COXIV (Proteintech, 66110-1-Ig,1:2500) at 4 °C overnight. Cells were washed 3 times with PBS and then incubated with Alexa Flours conjugated anti-rabbit or mouse IgG secondary antibodies (Invitrogen; 1:500) at room temperature for one hour. The numbers of Ki67 positive cells and total cells were manually counted from each microscopic field. The ratio of the number of Ki67 positive cells divided by the number of total cells from each field was calculated from 3 independent experiments. More than 5 fields for each group were analyzed in each experiment.

### Proximity ligation assay

PLA was performed on fixed A549 cells with DuoLink PLA technology probes and reagents (Sigma-Aldrich) following the manufacturer’s instructions. Briefly, cells (as specified in each experiment) were permeabilized with PBS containing 0.5% Triton X-100 for 15 min. After washing with PBS twice, the cells were incubated with blocking solution for 30 min at 37 °C and then incubated with pairs of rabbit anti-PYCR1 (Proteintech,13108-1-AP,1:200) and mouse anti-kindlin-2 (clone 3A3.5^60^, 1:500) or pairs of rabbit anti-PYCR1 (Proteintech,13108-1-AP,1:200) and mouse anti-FLAG (Sigma, F1804) antibodies as specified in each experiment at 4 °C overnight. The coverslips were washed 3 times with buffer A provided by the manufacturer for 5 min, followed by incubation with the PLA probes provided by the manufacturer in antibody diluent for 60 min at 37 °C. After washing three times with buffer A for 5 min, the ligation step was performed with ligase diluted in the ligation buffer for 30 min at 37 °C. The cells were washed twice with buffer A for 5 min, followed by incubation with amplification solution at 37 °C for 100 min. After washing twice with buffer B provided by the manufacturer for 10 min and once with 0.01×buffer B for 1 min, the coverslips were mounted with Duolink in situ mounting medium containing DAPI. A negative control was included in which one of the two antibodies was replaced with control IgG from the same species for all experiments.

### Immunoprecipitation

Cells (as specified in each experiment) were harvested and homogenized in IP lysis buffer (P0013, Beyotime, China) supplemented with 1 mM PMSF (Sigma-Aldrich, Cat# 329-98-6) for 30 min at 4 °C, and pre-cleared with Protein A/G PLUS-Agarose (Santa Cruz, Cat# sc-2003) for 30 min. IP was performed overnight at 4 °C by incubation of the cell lysates containing equal amount of proteins (0.5–2 mg) with mouse anti-kindlin-2 antibody (clone 3A3.5^60^) or irrelevant mouse IgG (Santa Cruz, Cat# sc-2025) (as a negative control) as specified in each experiment. Antibodies and associated proteins were immunoprecipitated by incubation of the samples with Protein A/G PLUS-Agarose for 2 h, followed by washing once with the lysis buffer and twice with PBS. The samples were then subjected to Western blotting using rabbit anti-PYCR1 (Proteintech,13108-1-AP,1:1000) or rabbit anti-kindlin-2 (Proteintech, 11453-1-AP,1:1000) antibodies. The integrated densities of the bands on Western blots (from at least three independent experiments) were quantified using Image J software (version 2.0.0-rc-69/1.52p, NIH, Bethesda, MD, USA).

### Live-cell imaging and analysis of mitochondrial morphology

Cells (as specified in each experiment) were incubated with 50 nM MitoTracker Red CMXRos (Life Technologies) in DMEM medium at 37 °C for 30 min. Mitochondria were visualized by leica TCS SP8 confocal microscopy under a 63× oil-immersion objective with a numerical aperture of 1.4 (zoom in 2.5x). Cells were kept on the stage at 37 °C with an incubation chamber equipped with a gas mixer and 5% humidified CO_2_. Images were collected on live cells using the Leica Biosystems Application Suite software package. Image size is 1024 × 1024 pixels.

Images of mitochondria were analyzed for length and width using *Surface* tool in the Imaris software (version 8.3.1). Specifically, we set the *geometry* option of the Imaris *Surface* tool (XYZ = 71.7 × 71.7 × 0.08 μm^3^ from the surface layer). Next, using t*he entire image* option of Imaris *Surface*, the same threshold parameters were set for mitochondria in each cell (surface detail = 0.036 μm; background subtraction diameter of largest sphere = 0.6 μm; split touching object point diameter=0.72μm. Manual adjustment of threshold was set to cover all mitochondria). Then, the surface length and width of mitochondria were collected and selected for statistical analyses. Mitochondria with different morphology (fragmented: length divided by width≤1.5; intermediate: length divided by widthå 1.5 ≤ 3.0 or elongated: length divided by width >3) were quantified. Sequential Z-stack series of mitochondria were obtained with the Leica Biosystems Application Suite software package with each Z-step at 0.8 mm. Mitochondrial 3D reconstruction was achieved using Surface tool of Imaris software (XYZ = 71.7 × 71.7 × 0.4 μm^3^ from the surface layer). The surface areas of mitochondria were collected and selected for statistical analyses. After individual mitochondrial area of each mitochondrial particle was obtained, the values were averaged for each cell. At least 30 cells from three independent experiments were analyzed.

### Transmission electron microscopy (TEM)

The cells were fixed with 2% glutaraldehyde, treated with 1% OsO4, alcohol dehydrated, and embedded in araldite. After staining with 3% uranyl acetate and 3% lead citrate, the sections were analyzed using a 120 kV compact-digital biological electron microscope (TEM-HT7700, Japan). Image J software (version 2.0.0-rc-69/1.52p, NIH, Bethesda, MD, USA) was used to quantify the mitochondrial areas.

### Analysis of cellular ATP level

The ATP levels in the cells were quantified using a commercially available luciferin-luciferase system (the ATP Assay Kit from Beyotime) following the manufacturer’s protocol. The cells were washed thoroughly in PBS and lysed with the lysis buffer provided by the ATP Assay Kit (Beyotime), followed by centrifugation at 10,000 × *g* for 2 min at 4 °C. The supernatants were transferred to fresh tubes and the levels of ATP were determined by luminometry using an EnSpire Multimode Plate Reader.

### Analysis of cellular NADPH/NADP ratio

The ratios of NADPH/NADP were analyzed using a NADPH/NADP Quantification Kit (Sigma-Aldrich, MAK038, UK) following the manufacturer’s protocol. The cells (4 × 10^6^ perassay) were washed with cold PBS, homogenized with 800 μL of NADPH/NADP Extraction Buffer, and centrifuged at 2000 rpm for 5 min at 4 °C. The supernatants were transferred to new tubes on ice. Total NADP (NADP and NADPH) and NADPH were quantified using a colorimetric assay (460 nm) with a microplate reader (Biotek, Epoch2, USA).

### Analysis of reactive oxygen species (ROS)

Cellular level of ROS was analyzed with Dihydroethidium (DHE) fluorescence probe (Beyotime, China) following the manufacturer’s protocol. Cells were seeded on fibronectin-coated coverslips in 24 well plates (2 × 10^4^ cells-per well) and cultured in the presence of vitamin C (100 μM), a water-soluble antioxidant, overnight. The cells were washed three times with PBS, labeled with DHE probe (10 μM), and incubated at 37 °C for 1 h. At the end of incubation, the cells were washed three times with PBS, and fixed in 4% paraformaldehyde at room temperature for 30 min. The cells were then washed three times with PBS and stained with Hoechst at room temperature for 15 min. The cells were observed under a fluorescent microscope (Olympus IX73, Olympus Co., Ltd.). The fluorescence intensities of more than five random microscopic fields at 20× magnification from each group were analyzed with Image J software (version 2.0.0-rc-69/1.52p, NIH, Bethesda, MD, USA).

### Analyses of cellular oxygen consumption rate (OCR)

Cellular respiration rates were performed using an XF24 flux analyzer (Seahorse Bioscience Inc. North Billerica, MA, USA) following the manufacturer’s instructions. The wild type or PINCH-1 KO A549 cells were plated at a density of 2.5 × 10^4^ cells per well in an XF-24-cell culture plate 24 h prior to the assay. Before measurements, cells were washed and incubated with XF DMEM Base Medium (BD) supplemented with 1 mM pyruvate, 2 mM glutamine, 10 mM glucose for 1 h at 37 °C, in the absence of CO_2_. OCR was measured under basal conditions followed by sequential injection of oligomycin (1 μM), an ATP synthase inhibitor, FCCP (1 μM), a mitochondrial oxidative phosphorylation uncoupler, and rotenone (Rot,0.5 μM) and antimycin A (AA, 0.5 μM), inhibitors for mitochondrial respiratory chain complex I and III, respectively. Basal oxygen consumption measurements were normalized by cell number.

### Measurement of proline level

The proline levels were analyzed using the ninhydrin method^11,62^. To do this, the cells (as specified in each experiment) were cultured with basic DMEM medium in the absence of FBS for 24 h. 4 × 10^6^ cells were lysed with PBST (1% Triton X-100 in PBS) and the cellular debris were removed by centrifugation (9000 × *g*). The supernatants were transferred to a boiling water bath, and intracellular amino acids were extracted by boiling for 10 min. After centrifugation (5 min, 4 °C, 15,000 × *g*), the proline level in the supernatant was determined^11,62^. To do this, 200 μL of the supernatant was incubated with 400 μL of 1.25% ninhydrin (0.125 g of ninhydrin dissolved in 6 mL of glacial acetic acid and 4 mL distilled water) for 20 min at 100 °C, followed by recording absorbance of the proline-ninhydrin condensation product in the reaction mixture itself at 508 nm. A standard curve ranging in concentrations of 0–500 ng ml^−1^ proline was generated and used for determination of proline concentrations of the samples. For measurement of proline level in the lung tissues, same lobes of the lung tissues from Kras^fl/+^ and Kras^fl/+^; P1^fl/fl^ mice were collected. The levels of proline in the lung tissues were quantified as described above.

We have also analyzed the proline level using ultra-performance liquid chromatography-tandem mass spectrometry (UPLC-MS/MS). To do this, the cells (4 × 10^6^) were cultured in 4 ml basic DMEM medium in the absence of FBS for 24 h. The cell culture supernatant (10 μl) from the cells (as specified in each experiment) was then transferred into a 2 ml centrifuge tube and mixed with 1000 µl of methanol containing 0.1% formic acid (v/v), vortexed for 30 seconds, and centrifuged at 12,000 rpm at 4 °C for 15 min. The supernatants (100 µl per tube) were then transferred into new Eppendorf tubes. After diluting 10 times with water, the samples (100 µl persample) were labeled with isotope (100 ppb) and vortexed for 30 seconds. The supernatants were filtered through 0.22 nm membrane and analyzed by UPLC-MS/MS. Chromatographic separation was accomplished in a Thermo Ultimate 3000 system equipped with an ACQUITY UPLC^®^ BEH C18 (100 × 2.1 mm, 1.7 µm, Waters). The mobile phase consisted of a mixture of A (10% methanol/90% water containing 0.1% formic acid (v/v)) and B (50% methanol/50% water containing 0.1% formic acid (v/v)). The column oven temperature was set at 40 °C with an injection volume of 5 µl. The gradient program was set as: 0–6.5 min, 10–30% B; 6.5–7 min, 30–100% B; 7–8 min, 100% B; 8–8.5 min, 100–10% B; 8.5–12.5 min, 10% B at the flow rate of 0.3 mL per min (0–8.5 min) and 0.3–0.4 mL permin (8.5–12.5 min). The ESI source was used in positive mode with the following conditions: temperature = 500 °C, ion spray voltage = 5500 V, collision gas = 6 psi, curtain gas = 30 psi, nebulizer gas = 50 psi and heater gas = 50 psi. The data analyses and quantitation were executed using the MRM (multiple reaction monitoring) mode.

### Analysis of collagen matrix

Collagen matrix in lung tissues was analyzed by second harmonic generation (SHG) with multiphoton microscopy (MPM)^63–66^. Mouse lung tissues (as specified in each experiment) at 16 weeks after Ad-Cre infection were isolated, fixed in 10% formalin, and embedded in paraffin as described^60^. Sections (5 μm thick) were de-paraffinized and re-hydrated. Then, thin lung sections were mounted on coverslips with Neutral Balsam (Yeasen, 36313ES60) for MPM. MPM was performed with a FVMPE-RS MPM system (Olympus, Japan) based on a Olympus IX83 inverted microscope, which was equipped with a femtosecond-pulsed Ti:Sa laser (Mai Tai DeepSee, Spectra-physics, USA). Emitted signals were collected using an apochromat objective (×20/NA 0.75/WD 0.6, Olympus, Japan), and detected with two non-descanned photomultipliers and one camera (transmission detector, TD). When imaging, the excitation laser was tuned to 960 nm and collagen matrix in lung sections was visualized by SHG of the excitation laser in backscattering mode. Emission signals were separated by a dichroic mirror and two band pass filters (505DCXR, 480/40, 540/40 respectively, Chroma Technology, USA). Collagen signals by SHG was collected in the 480/40 channel while the 540/40 channel was to record autofluorescence (AF) as result of sample preparation. Merged SHG/AF images were generated to identify collagen signals. The mean intensities of collagen were quantified using Image J.

In some experiments, the collagen matrix in lung tissues was also stained with Masson’s trichrome staining (Solarbio Life Science: G1340). The areas of Masson’ trichrome positive regions were analyzed with Image J (version 2.0.0-rc-69/1.52p, NIH, Bethesda, MD, USA). For each experimental group, six sections of lung tissues from 6 individual mice were used and more than 5 fields for each section were analyzed.

### Statistical analysis

Data are presented as means ± SEM. Statistical analysis was performed using Student’s *t* test (two-tailed) or one-way ANOVA with Tukey’s post-hoc test by GraphPad Prism (version 7). Survival functions were plotted using the Kaplan–Meier method, and comparison of survival functions was performed by the log-rank test. A *p* value < 0.05 was considered significant. Statistical parameters including statistical significance and n value are shown in the figures or figure legends.

### Reporting summary

Further information on research design is available in the Nature Research Reporting Summary linked to this article.

## Supplementary information

Supplementary Information

Supplementary Data 1

Reporting Summary

## Data Availability

All data generated or analyzed during this study are included in this published article (and its Supplementary information files). The source data underlying Figs. 1–10 and Supplementary Figs. 1, 2, 4–7 are provided as a Source Data file. Source data are provided with this paper.

## Source data

Source Data
