# Supplementary material for: PINCH-1 regulates mitochondrial dynamics to promote proline synthesis and tumor growth
Source: Nat Commun. 2020 Oct 1;11:4913. doi: 10.1038/s41467-020-18753-6 (PMC7529891; doi:10.1038/s41467-020-18753-6)
Supplement: Supplementary file 1 — Supplementary Information [file 41467_2020_18753_MOESM1_ESM.pdf]

PINCH-1 regulates mitochondrial dynamics to promote proline synthesis and tumor growth

Guo et al.

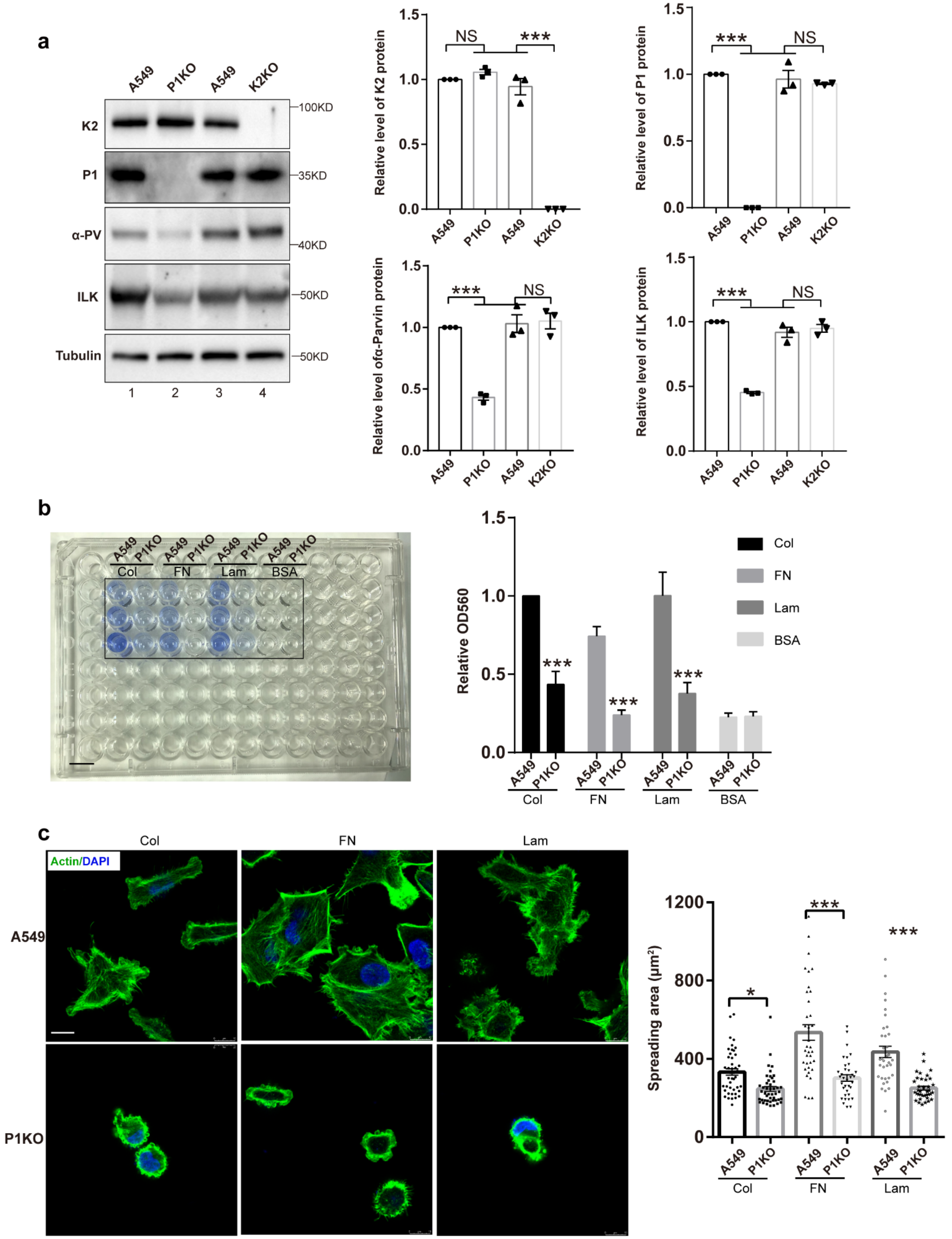

supplementary Fig.1

Supplementary Figure 1. The effects of PINCH-1 deficiency on ILK, α-parvin and kindlin-2 levels, cell-ECM adhesion and spreading

(a) PINCH-1 KO (P1KO) or kindlin-2 KO (K2KO) A549 cells were analyzed by Western blotting with antibodies recognizing K2, P1, α-parvin, ILK or tubulin as indicated. The levels of K2, P1, α-parvin (α-PV) and ILK in P1KO or K2KO A549 cells were quantified by densitometry and compared to those in A549 cells (normalized to 1) (right, n=3; K2 (A549 vs P1KO) P=0.6434, K2 (A549 vs K2KO) P<0.0001, P1 (A549 vs P1KO) P<0.0001, P1 (A549 vs K2KO) P=0.4722, α-parvin (A549 vs P1KO) P=0.0002, α-parvin (A549 vs K2KO) P=0.8803, ILK (A549 vs P1KO) P<0.0001, ILK (A549 vs K2KO) P=0.5112). The samples were from the same experiment and the blots were processed in parallel. (b) The adhesion of wild type or PINCH-1 KO A549 cells to collagen I, fibronectin, laminin-1 or BSA as a control was analyzed as described in the "Methods". Scale bar =1cm; n = 3; Col (A549 vs P1KO) P=0.0002, FN (A549 vs P1KO) P=0.0005, Lam (A549 vs P1KO) P<0.0001. (c) PINCH-1 KO A549 cells were immunofluorescently stained with Alexa Fluor–phalloidin (green) and DAPI (blue). Scale bar =25μm. Cell spreading was analyzed as described in the "Methods" (right, Col n=45 cells, FN n=36 cells, Lam n=36 cells; Col (A549 vs P1KO) P=0.0364, FN (A549 vs P1KO) P<0.0001, Lam (A549 vs P1KO) P<0.0001). Col: collagen I; FN: fibronectin; Lam: laminin-1. Data represent mean ± SEM. Statistical significance was calculated using one-way ANOVA with Tukey–Kramer post-hoc analysis (a) or two-tailed unpaired Student's t-test (b, c), *P < 0.05; ***P < 0.001; NS, no significance. Source data are provided as a Source Data file.

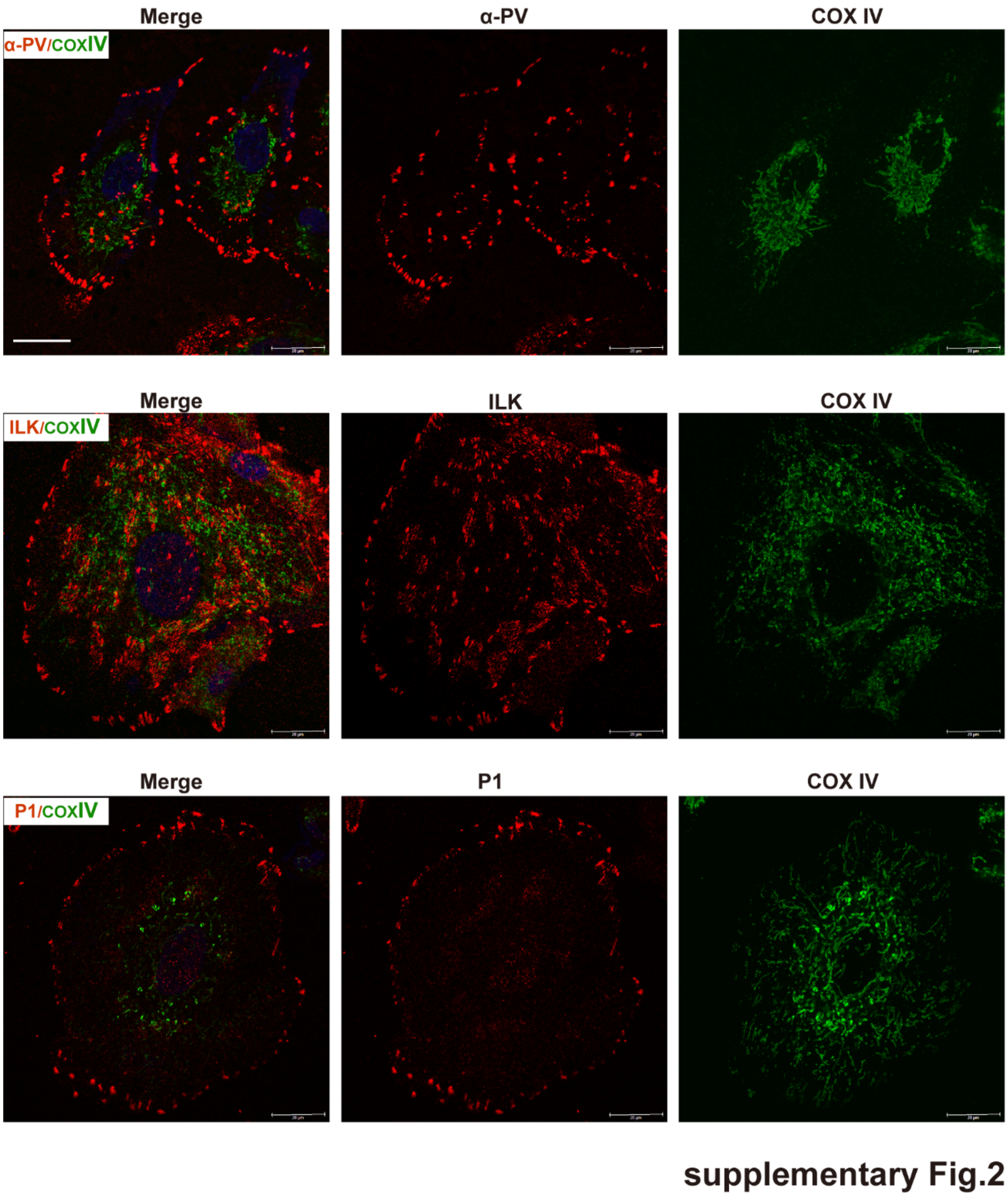

Supplementary Figure 2. Immunofluorescent staining of PINCH-1, ILK and α-parvin

A549 cells were immunofluorescently stained with antibodies for CoxIV (green), a marker of mitochondria, and PINCH-1 (red), α-Parvin (red) or ILK (red) as indicated. Scale bar = 20 μm. Three independent experiments were performed and similar results were obtained. Images from a representative experiment are shown.

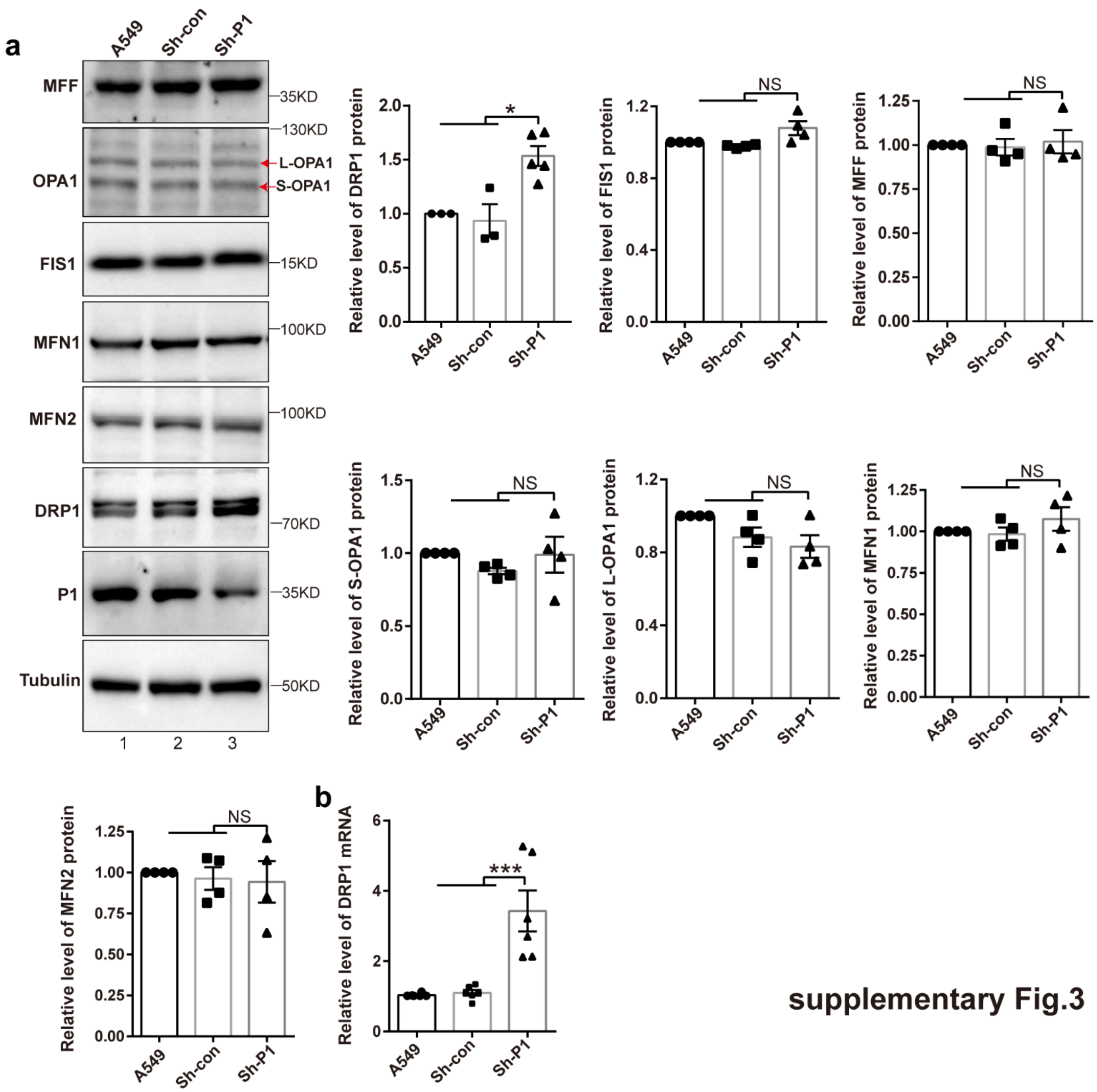

Supplementary Figure 3. Knockdown of PINCH-1 increases DRP1 expression

(a) A549 cells were infected with PINCH-1 shRNA or control RNA lentivirus, cultured in normal medium for 5 days and analyzed by Western blotting with antibodies recognizing DRP1, FIS1, MFF, OPA1, MFN1, MFN2 or tubulin as indicated. The levels of DRP1, FIS1, MFF, S-OPA1, L-OPA1, MFN1 and MFN2 in the infected cells were quantified by densitometry

and compared to those in uninfected cells (normalized to 1) (right, A549 n=3, Sh-con n=3, Sh-P1 n=5; DRP1 (A549 vs Sh-P1) P=0.014, DRP1 (Sh-con vs Sh-P1) P=0.0076). (b) The mRNA levels of DRP1 were analyzed by RT-PCR (n=6; A549 vs Sh-P1 P=0.0005, Sh-con vs Sh-P1 P=0.0006). The samples were from the same experiment and the blots were processed in parallel. Data present mean ± SEM using one-way ANOVA with Tukey–Kramer post-hoc analysis, *P < 0.05; ***P < 0.001; NS, no significance. Source data are provided as a Source Data file.

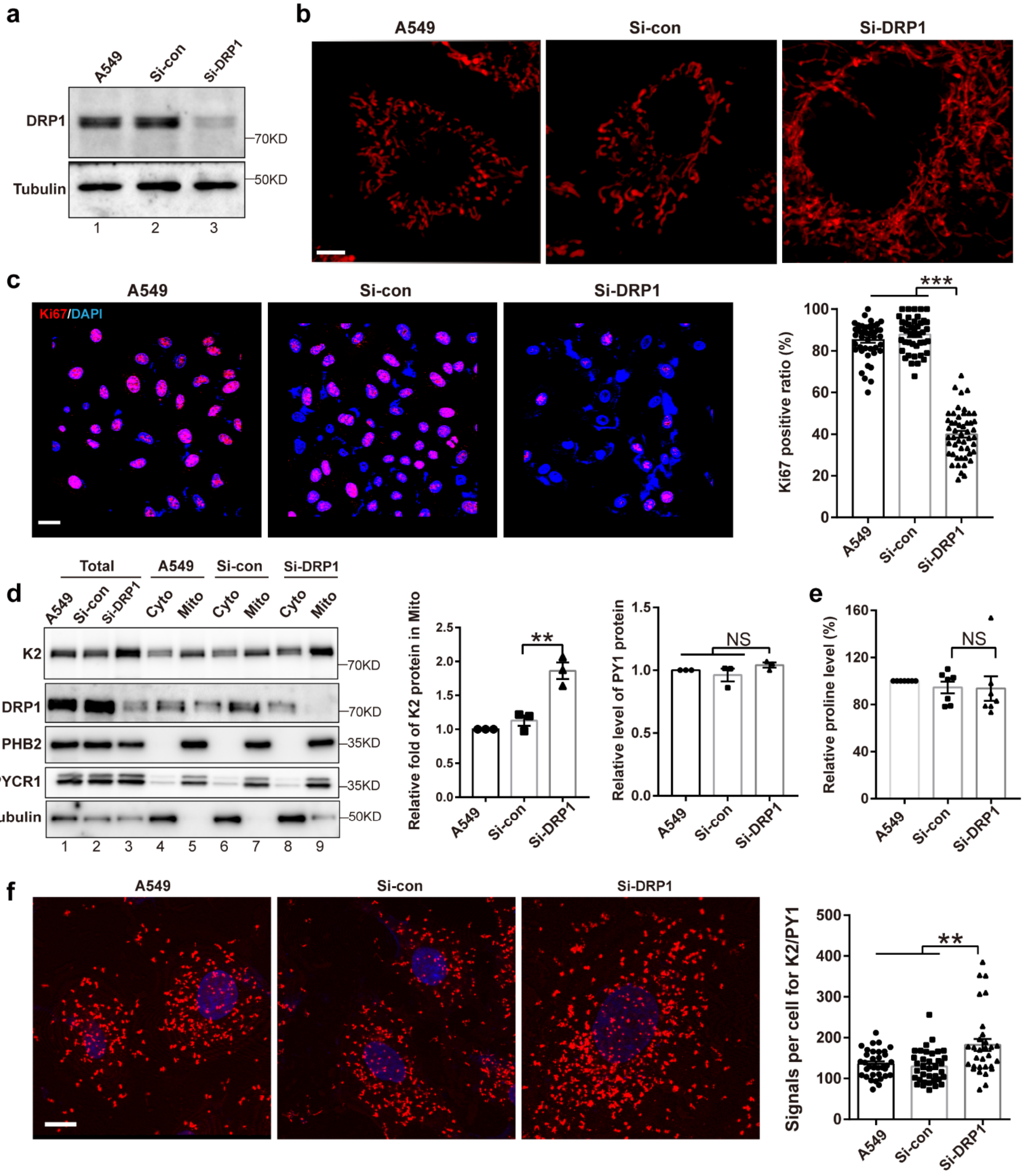

supplementary Fig.4

Supplementary Figure 4. The effects of depletion of DRP1 from PINCH-1 expressing A549 cells

(a) A549 cells were transfected with Si-DRP1 or Si-con as indicated. Three days later, cells (as indicated) were analyzed by Western blotting with DRP1 and tubulin antibodies. (b) Mitochondrial morphology was analyzed by MitoTracker Red CMXRos. Bar, 5μm. (c) Cells were stained with DAPI and anti-Ki67 antibody. Bar, 25μm. The percentages of Ki67-positive cells were quantified (right, A549 n=45 fields, Si-con n=44 fields, Si-DRP1 n=50 fields; A549 vs A549+Si-DRP1 $P<0.0001$, A549+Si-con vs A549+Si-DRP1 $P<0.0001$). (d) The cytosolic (lane 4,6 and 8), mitochondrial (lane 5, 7 and 9) and total (lane 1, 2 and 3) fractions from the cells were analyzed by Western blotting with antibodies recognizing kindlin-2, PHB2, DRP1, PYCR1 or tubulin (left). Middle, the levels of kindlin-2 in the cytosolic or mitochondrial fractions were quantified by densitometry and the ratio of mitochondrial kindlin-2 level divided by that of cytosolic kindlin-2 level in the DRP1 knockdown cells was compared to that in the control transfectants or wild type A549 cells (normalized to 1, right, n=3; $P=0.002$). Left, the PYCR1 level in the DRP1 knockdown cells was quantified and compared to that in the control transfectants or wild type A549 cells (right, n=3; $P=0.2577$). (e) The proline level was analyzed using the absorbance method as described in the “Methods” (n= 7 independent experiments; $P=0.9951$). (f) The cells were analyzed by PLA with kindlin-2 and PYCR1 antibodies. Bar, 10μm. The numbers of PLA dots per cell were counted (right; A549 n=34 cells, Si-con n=36 cells, Si-DRP1 n=30 cells; A549 vs Si-DRP1 $P=0.0025$, Si-con vs Si-DRP1 $P=0.0004$). Data represent mean ± SEM. Statistical significance was calculated using one-way ANOVA with Tukey–Kramer post-hoc analysis, $^{**}P < 0.01$; $^{***}P < 0.001$; NS, no significance. Source data are provided as a Source Data file. The samples in a and d were from the same experiment and the blots were processed in parallel.

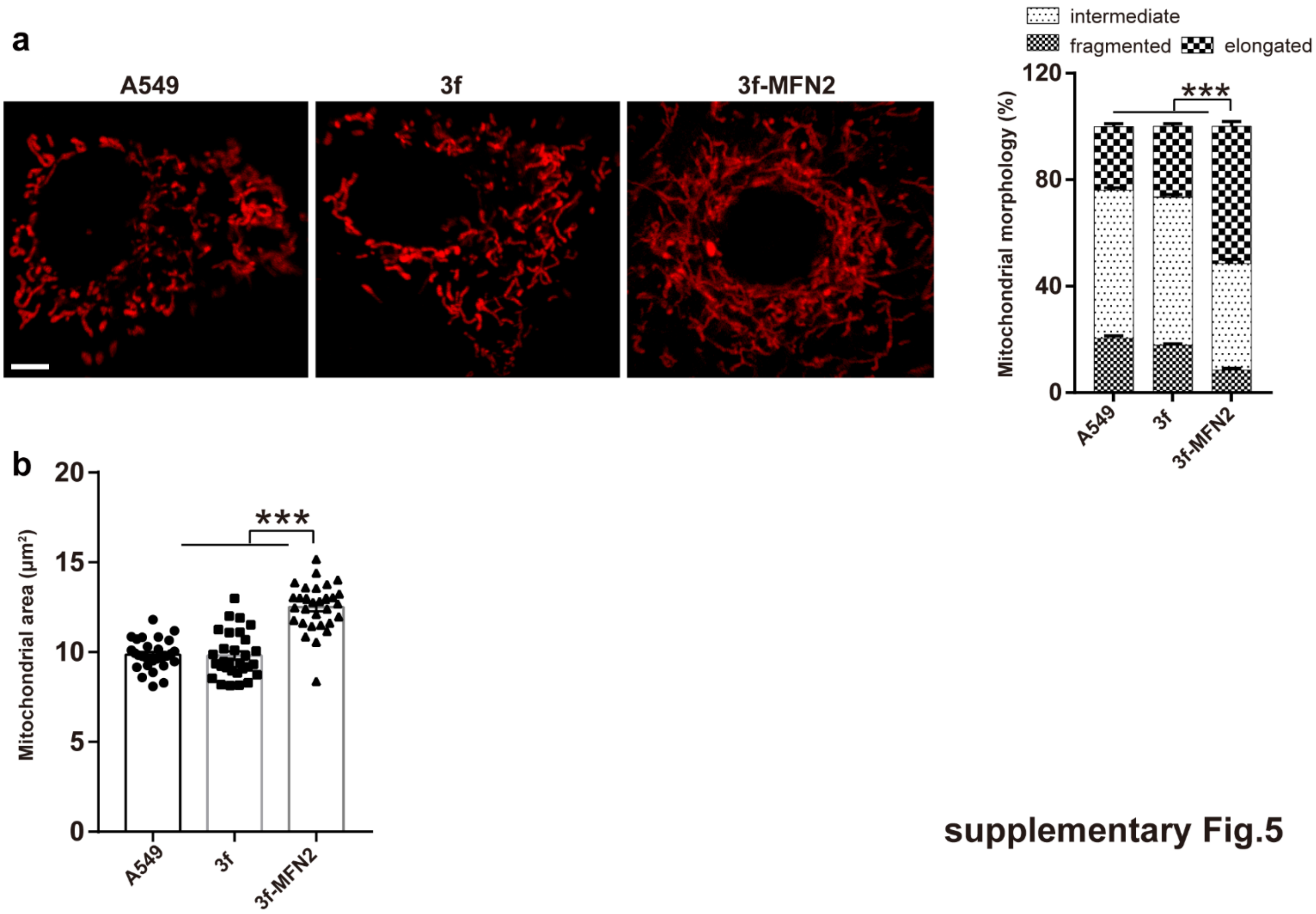

supplementary Fig.5

Supplementary Figure 5. The effects of overexpression of MFN2 on mitochondrial morphology

A549 cells were infected with lentiviral vectors encoding 3f-MFN2 or 3f only as a control. Three days later, mitochondria were stained with MitoTracker Red CMXRos and mitochondria with different morphologies were quantified (n=30 cells; $P<0.0001$) (a). Bar, 5μm. Mitochondrial areas in z-stack images were quantified (n=30 cells; $P<0.0001$) (b). Data represent mean ± SEM. Statistical significance was calculated using one-way ANOVA with Tukey–Kramer post-hoc analysis, ***$P < 0.001$. Source data are provided as a Source Data file.

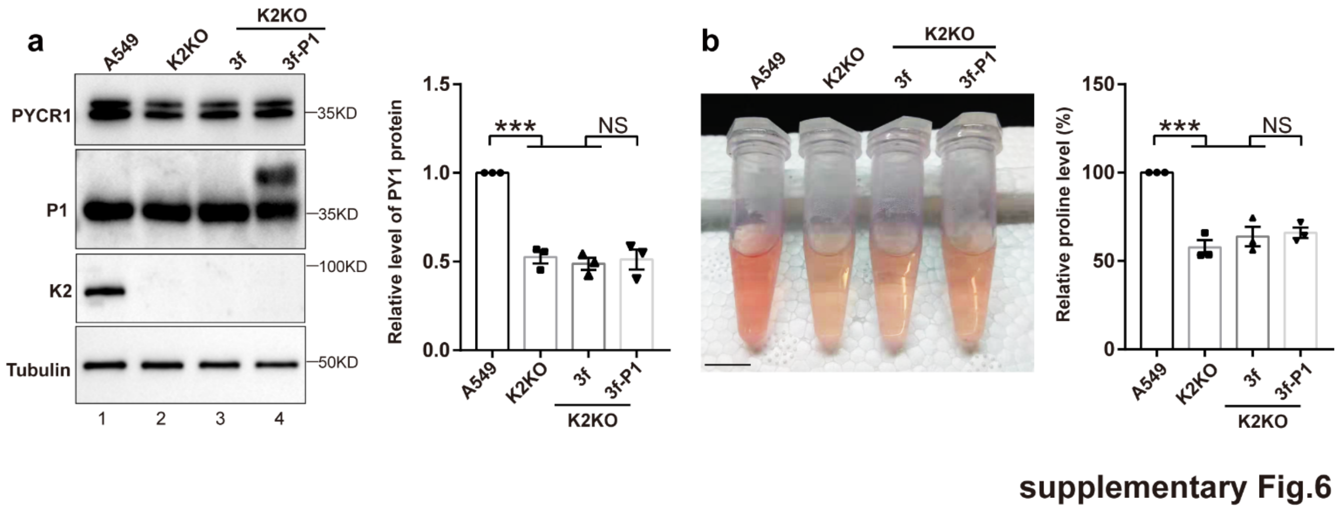

Supplementary Figure 6. Overexpression of PINCH-1 fails to increase the levels of PYCR1 and proline in the absence of kindlin-2

Kindlin-2 KO A549 cells were infected with lentiviral vectors encoding 3f-P1 or 3f. Three days later, the cells were analyzed by Western blotting with antibodies recognizing K2, PYCR1, P1 or tubulin (a, left). The PYCR1 protein levels in the cells (as indicated) were quantified by densitometry and compared to that in A549 cells (a, right) (n=3; A549 vs K1KO P<0.0001, A549 vs KO2KO+3f P<0.0001, K2KO vs K2KO+3f-P1 P=0.9926, K2KO+3f vs K2KO+3f-P1 P=0.9674). The samples were from the same experiment and the blots were processed in parallel. (b) The proline level was analyzed using the absorbance method as described in the "Methods" (right, n=3 independent experiments; A549 vs K1KO P=0.0002, A549 vs KO2KO+3f P=0.0006, K2KO vs K2KO+3f-P1 P=0.4495, K2KO+3f vs K2KO+3f-P1 P=0.9774). A representative set of samples were shown in the left. Bar, 1cm. Data represent mean ± SEM. Statistical significance was calculated using one-way ANOVA with Tukey–Kramer post-hoc analysis, ***P < 0.001; NS, no significance. Source data are provided as a Source Data file.

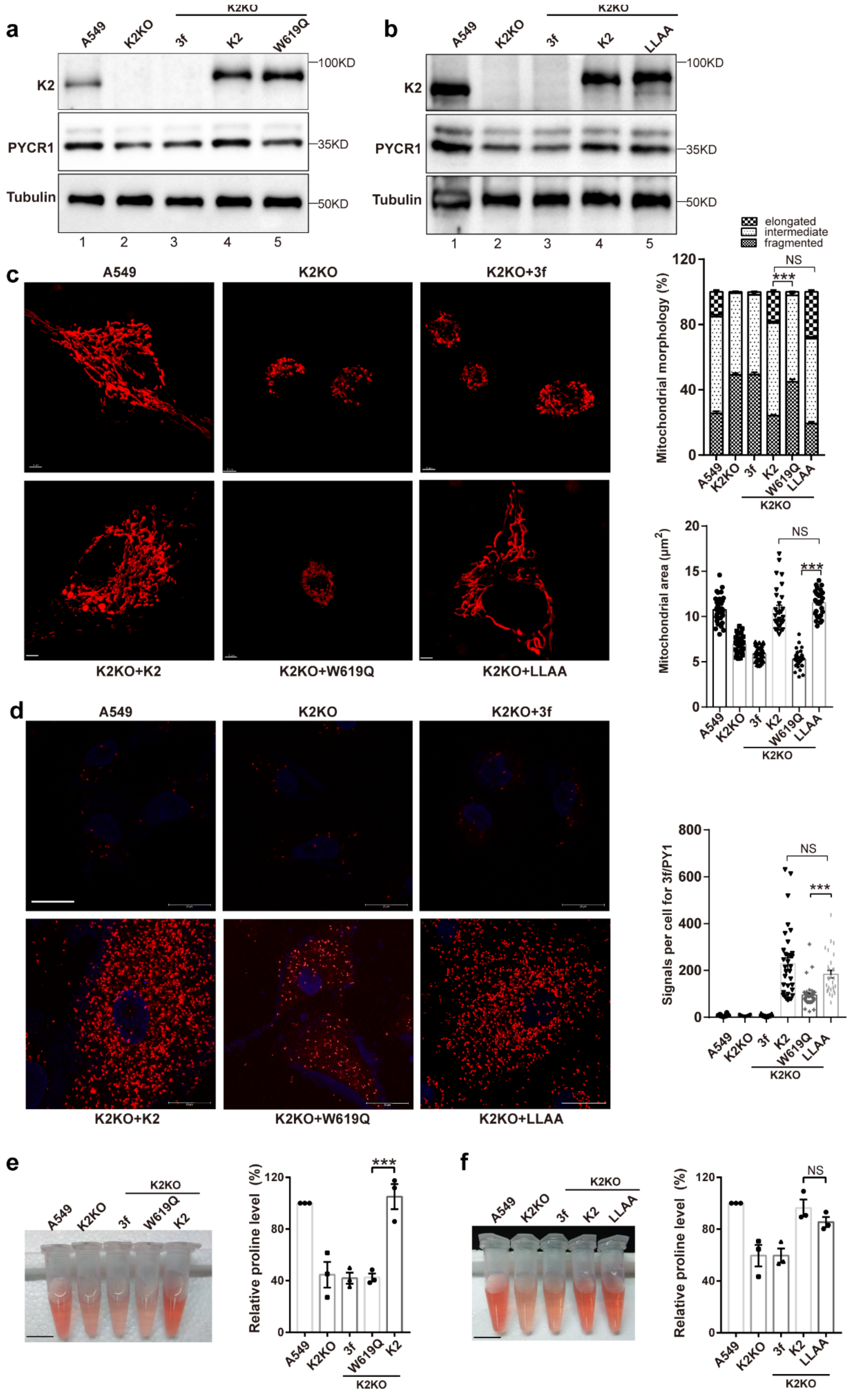

supplementary Fig.7

Supplementary Figure 7. The effects of W619Q and L353A/L357A mutations on kindlin-2 interaction with PYCR1 and proline synthesis

Kindlin-2 KO cells were infected with lentiviral vectors encoding 3xFLAG-tagged kindlin-2, 3xFLAG-tagged kindlin-2 W619Q mutant, 3xFLAG-tagged kindlin-2 L353A/L357A (LLAA) mutant or 3xFLAG vector lacking kindlin-2 sequence (3f) as a control. Three days later, the cells were analyzed by Western blotting (a and b). (c) Mitochondrial was stained with MitoTracker Red CMXRos (left panels). Bars, 5μm. Mitochondria with different morphologies were quantified (the top panel on the right, n=30 cells; K2KO+3f-K2 vs K2KO+3f-K2 (W619Q) P<0.0001). Mitochondrial areas in z-stack images were quantified (the bottom panel on the right, n=30 cells; K2KO+3f-K2 (W619Q) vs K2KO+3f-K2 (LLAA) P<0.0001). (d) The cells were analyzed by PLA with FLAG and PYCR1 antibodies. Bar, 20μm. The numbers of PLA dots per cell were counted (right, A549 n=40 cells, K2KO n=36 cells, K2KO+3f n=35 cells, K2KO+3f-K2 n=34 cells, K2KO+3f-K2 (W619Q) n=37 cells, K2KO+3f-K2 cells n=30 cells; K2KO+3f-K2 (W619Q) vs K2KO+3f-K2 (LLAA) P<0.0001). (e and f) The proline levels in the cells (as specified in the figure) were analyzed using the absorbance method as described in the “Methods”. Representative sets of samples were shown in the left. Bar, 1cm. The data in the right panels represent mean ± SEM (n=3 independent experiments; K2KO+3f-K2 (W619Q) vs K2KO+3f-K2 P=0.0004, K2KO+3f-K2 vs K2KO+3f-K2 (LLAA) P=0.6491). Statistical significance was calculated using one-way ANOVA with Tukey–Kramer post-hoc analysis, ***P < 0.001; NS, no significance. Source data are provided as a Source Data file.

Supplementary table 1. List of primers used for quantitative RT-PCR

Gene	Sequence (5' to 3')	
GAPDH (h)	Sense	CCAGAACATCATCCCTGCCTCTACT
	Antisense	GGTTTTTCTAGACGGCAGGTCAGGT
DRP1(h)	Sense	GTAGGCCAGCTGTATAAATCATCC
	Antisense	TTGTAATGCCTTTAGCATATCAGC
GAPDH (m)	Sense	TACAGCAACAGGGTGGTGGAC
	Antisense	TGGGATAGGGCCTCTCTTGCT
DRP1(m)	Sense	TAAGCTGCAGGACGTCTTCA
	Antisense	GAAACGTGGACTAGCTGCAG
